# Symmetric Projection Attractor Reconstruction: Sex Differences in the ECG

**DOI:** 10.3389/fcvm.2021.709457

**Published:** 2021-09-23

**Authors:** Jane V. Lyle, Manasi Nandi, Philip J. Aston

**Affiliations:** ^1^Department of Mathematics, University of Surrey, Guildford, United Kingdom; ^2^School of Cancer and Pharmaceutical Sciences, Faculty of Life Sciences & Medicine, King's College London, London, United Kingdom

**Keywords:** electrocardiogram, sex and gender, patient stratification, ECG waveform analysis, symmetric projection attractor reconstruction, machine learning

## Abstract

**Background:** The electrocardiogram (ECG) is a key tool in patient management. Automated ECG analysis supports clinical decision-making, but traditional fiducial point identification discards much of the time-series data that captures the morphology of the whole waveform. Our Symmetric Projection Attractor Reconstruction (SPAR) method uses all the available data to provide a new visualization and quantification of the morphology and variability of any approximately periodic signal. We therefore applied SPAR to ECG signals to ascertain whether this more detailed investigation of ECG morphology adds clinical value.

**Methods:** Our aim was to demonstrate the accuracy of the SPAR method in discriminating between two biologically distinct groups. As sex has been shown to influence the waveform appearance, we investigated sex differences in normal sinus rhythm ECGs. We applied the SPAR method to 9,007 10 second 12-lead ECG recordings from Physionet, which comprised; Dataset 1: 104 subjects (40% female), Dataset 2: 8,903 subjects (54% female).

**Results:** SPAR showed clear visual differences between female and male ECGs (Dataset 1). A stacked machine learning model achieved a cross-validation sex classification accuracy of 86.3% (Dataset 2) and an unseen test accuracy of 91.3% (Dataset 1). The mid-precordial leads performed best in classification individually, but the highest overall accuracy was achieved with all 12 leads. Classification accuracy was highest for young adults and declined with older age.

**Conclusions:** SPAR allows quantification of the morphology of the ECG without the need to identify conventional fiducial points, whilst utilizing of all the data reduces inadvertent bias. By intuitively re-visualizing signal morphology as two-dimensional images, SPAR accurately discriminated ECG sex differences in a small dataset. We extended the approach to a machine learning classification of sex for a larger dataset, and showed that the SPAR method provided a means of visualizing the similarities of subjects given the same classification. This proof-of-concept study therefore provided an implementation of SPAR using existing data and showed that subtle differences in the ECG can be amplified by the attractor. SPAR's supplementary analysis of ECG morphology may enhance conventional automated analysis in clinically important datasets, and improve patient stratification and risk management.

## Introduction

The electrocardiogram (ECG) plays a key role in the diagnosis, treatment and monitoring of cardiovascular disease. Qualitative analysis of the ECG by clinicians incorporates the morphology of the whole waveform, but much of the automated analysis undertaken to support rapid clinical decision-making focuses on the identification of fiducial points on the signal, and discards the intervening data. Although there is an increasing emphasis on waveform morphology within ECG research, this still tends to be concentrated on specific parts of the signal (1, 2).

Early studies into the nature of the ECG of healthy individuals identified various factors that impact the ECG, including sex, age and body shape (3–6). Whilst such differences may be appreciated qualitatively at a clinical level, they are not routinely incorporated into the analysis or interpretation of ECG parameters (7). However, with the increasing automated analysis of the ECG to support clinical decision-making and the application of machine learning to problems such as risk stratification, there is a renewed focus on factors that may have a subtle impact on an individual's ECG.

We have recently developed an approach to signal analysis that allows us to easily quantify information from the morphology of the whole waveform using all of its underlying high fidelity time-series data. Symmetric Projection Attractor Reconstruction (SPAR) is a method for visualizing and quantifying the morphology and variability of any approximately periodic signal (8–10), effectively replotting all the underlying data into a simpler two-dimensional representation, which we call an ‘attractor’. We have previously applied SPAR to various physiological signals, including the ECG (11, 12), arterial blood pressure (8–10, 13) and photoplethysmogram (PPG) (14), where it has been shown to supplement standard cardiovascular assessment.

Our primary motivation for this study was to demonstrate the accuracy of SPAR in discriminating between two biologically distinct groups. Significant sex differences have been demonstrated in the ECG through the conventional assessment of fiducial points, including a reduced QRS amplitude and longer QT interval in females, which are proposed to result from the interaction of various factors, including an individual's anatomy, endocrinology, autonomic regulation, and genetics (15, 16). Therefore, we focused on a binary discrimination of sex in resting ECGs, selecting signals that had been pre-adjudicated as ‘normal sinus rhythm’ to avoid introducing further confounding factors due to disease states.

Recent studies (17, 18) have applied deep learning techniques to problem of sex classification from the ECG, and showed that sex can be discriminated with high accuracy by combining all 12 leads. However, the ‘black box’ nature of the methods in these studies does not readily provide an insight into how these results are achieved. Recognizing that a clinical audience welcomes more clarity around how such machine learning classification results are generated and how they can be interpreted, we focused this study on the development of visualization tools within the SPAR approach and incorporated a classification of sex by lead to facilitate the interpretation of our results in the context of the existing literature.

We applied the unique visualization of the SPAR method to the ECG in a two part study using publicly available 10 second 12-lead ECGs from Physionet (19). In the first part, we introduced our approach using Dataset 1 (104 subjects) and showed how our attractor images corresponded to known characteristics of sex differences in the ECG. We then extended our study to Dataset 2 (8,903 subjects) and showed how SPAR can be applied to the problem of stratifying a larger population using machine learning, and how the visualization tools of SPAR analysis support the interpretation of our findings.

## Methods

**Figure 1:**
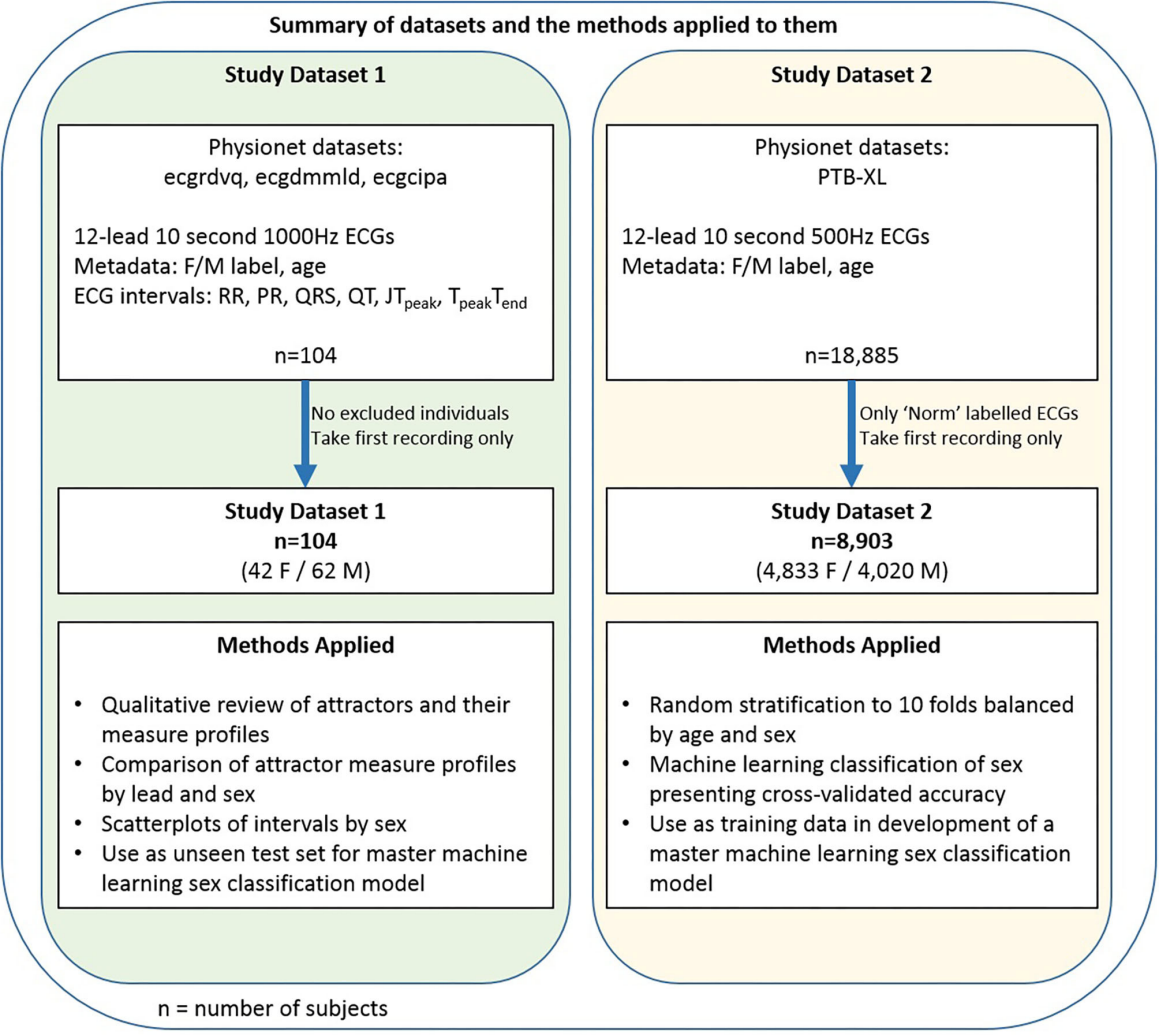
Data and methods summary. A summary of the two datasets and an overview of the methods applied to them.

We undertook a two part study. Figure 1 provides an overview of the dataset and methods applied in each arm of the study. Further details on all parts of the Methods are included in the Supplementary Material.

### Data

The data for each part of the study was denoted Dataset 1 and Dataset 2 respectively. For both parts, publicly available 10 second 12-lead ECGs were obtained from Physionet (19). These databases are well documented and further information can be found in the references given below. All subjects had a sex label (Female/Male) provided in the accompanying metadata. Only the first recording was taken for any subject if multiple recordings were available.

Dataset 1 consisted of the first baseline 12-lead 1000Hz ECG recordings from all 104 subjects (42 female, 19–50 years) who participated in the ecgrdvq, ecgdmmld and ecgcipa experimental studies (2, 20–22). Each study involved healthy adults, with baseline ECG recording taken prior to a pharmacological challenge. Validated standard ECG interval data (RR, PR, QRS, QT) and more nuanced repolarisation intervals (JT_peak_ and T_peak_T_end_) were provided in the accompanying metadata.

Dataset 2 comprised the 12-lead 500Hz ECG recordings of 8,903 subjects (4,833 female, 2–94 years) from the PTB-XL database (23) where the ECG recording was labeled “Norm” (noting that the alternative labels to “Norm” were “Myocardial Infarction”, “ST/T Change”, “Conduction Disturbance” or “Hypertrophy”). This label criteria identified 9,528 recordings since some subjects had more than one recording, but we took only the first recording labeled “Norm” from each subject. We also note that the label “Norm” reflects a normal sinus rhythm appearance on the ECG, which may not necessarily mean that the subject was healthy, and a subject may have had a separate prior recording which was not deemed to be of normal appearance.

All the ECG recordings were short, so each 10 second signal was analyzed as a single window. Importantly, no further filtering was applied to the signals taken from Physionet and no data was discarded.

### SPAR Method: Attractor Generation

**Figure 2:**
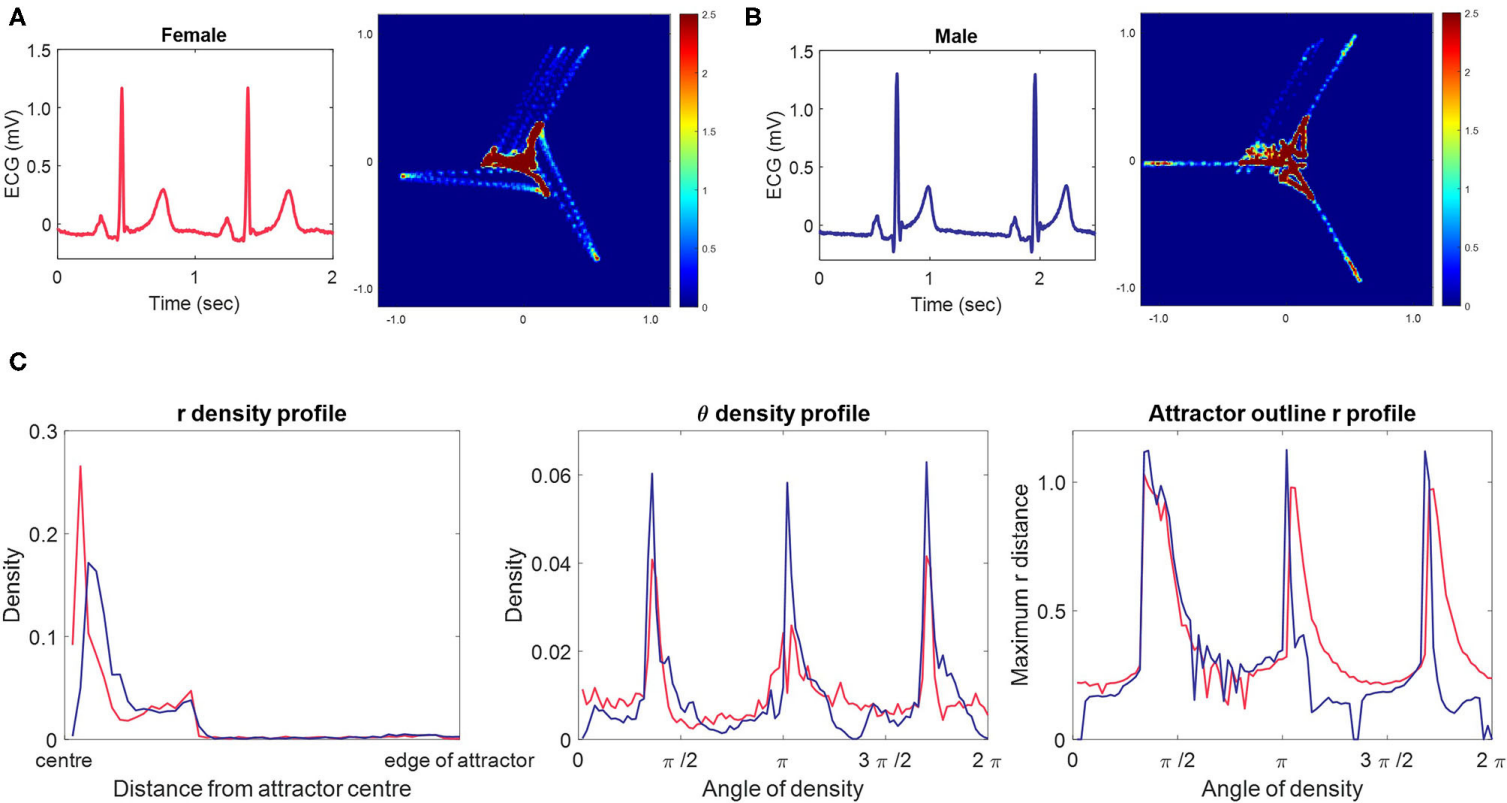
Typical female and male ECG signals, their corresponding 3-point attractors and attractor measure set profiles. **(A, B)** A 2 second excerpt from 10 second lead II signals from a typical female **(A)** and male **(B)** lead II signal with their corresponding 3-point attractors. Small differences in the appearance of the signals are amplified in the attractor. The overall appearance of the female attractor is one of a more dispersed distribution with wider arms, whereas the male attractor shows higher density, narrow arms. We also observe that the upper arm of both attractors is more variable than the other two arms. For both subjects, this is the result of the beat-to-beat variation in the T to P (interbeat) interval. **(C)** The measure set profiles associated with the attractor images in **(A)** [female, red] and **(B)** [male, blue.] The three measure sets quantify the attractor images, and we can clearly see differences between the female and male measures. The r density profile shows that both attractors have their points concentrated in the first third of the attractor moving out from the center, but that the density peak is more central and greater in the female attractor. The θ density indicates that although each attractors most dense parts lie at the same angle from the center, they are of lower density in the female attractor. The attractor outline r profile indicates that the male attractor is slightly larger than the female one and highlights that both attractors reflect beat-to-beat variability in their upper arm.

The SPAR method transforms the entire digital signal into a corresponding two-dimensional image. Our original method (8) places *N* = 3 equally spaced points on the signal. As these points are moved along the signal, their values are plotted in three-dimensional phase space, creating a bounded representation of the entire waveform. We then project the resulting three-dimensional object to a two-dimensional image, which we call an “attractor”. Figures 2A,B shows two ECG signals and their corresponding attractors, and we encourage those unfamiliar with the method to view the Supplementary Videos I, II, which show how the signal is transformed into the attractor.

To date, our SPAR method has used *N* = 3 points on the signal as described previously (8). However, there is no constraint that we have to take only three points on the signal, and any number of points could be used, allowing different waveform features to be emphasized whilst still providing a simple two-dimensional ‘attractor’ visualization. The use of *N* > 3 points is particularly pertinent for a complex waveform such as the ECG where the different features of the signal's morphology represent distinct biological processes. Increasing the value of *N* should enable the amplification of more features from the waveform and we have therefore extended our SPAR method to such cases (24) (also, Lyle JV, Aston PJ, submitted). Thus we investigated the use of different numbers of points in this study and found that complementary information was provided from attractors generated with all odd numbers of points for *N* = 3, 5, …, 13. Supplementary Figure 2 provides further information on the generation of attractors with *N* > 3 points. We will refer to an ‘*N*-point attractor’ to clarify the number of points used in its generation.

The SPAR method requires an equal spacing between the *N* points placed on the signal. This spacing is taken as 1/*N* of the average cardiac cycle length of the signal. For example, if the average heart rate of the signal is 60 beats per minute, then the average cycle length is 1 second and the points for a 3-point attractor will be placed 1/3 second apart. We determined the average cardiac cycle length of the signal using a QRS detector based on the Pan-Tompkins method (25, 26).

Each lead of a 12-lead ECG has a different appearance and so has its own corresponding attractor. We therefore generated attractors using *N* = 3, 5, …, 13 points from each lead of every signal in Dataset 1 and Dataset 2.

### SPAR Method: Quantifying the Attractor

Whilst the attractor provides a simple visualization of the ECG, it also allows for its features to be easily quantified. An image on a plane can be described by its polar coordinates, the radial distance r and angular distance θ, and we take this approach to generate three sets of measures that summarize the attractor:

- the radial density distribution (r density) based on the distance from the center to the outer edge of the attractor,
- the θ density distribution (θ density), and
- the outline shape of the image as maximum r in the θ direction (attractor outline r).

These measures are illustrated by measure profile plots in Figure 2C. Further details on how these measure sets are determined are provided in the Supplementary Material and in Supplementary Figure 3.

### Machine Learning: Binary Classification of Sex Using the Attractor Measures

The attractor image allows us to quantify the morphology and variability of the underlying signal, with small changes on the waveform being amplified on the corresponding attractor. To provide a proof-of-concept assessment of the clinical utility of the ECG attractor, we used machine learning as a tool for recognizing patterns in the attractor measures sets (r density, θ density, attractor outline r) and made a simple binary classification of sex from these.

#### Development of the Machine Learning Model

We developed our machine learning model using Dataset 2. Given the size of the dataset (8,903 records) we opted to present a cross-validated result, and Dataset 2 was initially randomly stratified into 10 folds (*n* = 890 or *n* = 891) such that data was balanced by sex and age (over 10 year ranges) in each fold. The folds used can be found within the accompanying files of the Supplementary Material. To ensure independence of model development and optimization, each cross-validation run separated training, validation and test folds. When we introduced a stacked model (as discussed below), this independence was maintained by a nesting of the cross-validation. The Supplementary Material and Supplementary Figure 4 describe the detail of this approach.

The early phases of our machine learning investigation for this study were exploratory to determine the most appropriate techniques. The high inter-individual variability of the normal ECG and the numerous factors that are known to impact its appearance (16) suggest that differences will be subtle and that we need to build up a model from relatively weak predictors. Using this and our previous experience in machine learning on the attractor (11, 12, 27), we started by applying a *k*-nearest neighbors (*k*-NN) algorithm to provide a classification of sex for each measure set (r density, θ density, attractor outline r) for each attractor.

Our preliminary results indicated that the use of a stacked model (28), whereby the outcome of separate classifications were then combined in a second classifier for either the lead or the subject was the most successful (when measured by the accuracy of classification). We also determined that the use of the posterior probability scores of each separate classification provided a more useful input to the second classifier than a binary Female / Male label. At different stages of the preliminary investigation, we considered different machine learning algorithms as both the first or second classifier, including support vector machines (SVM), random forest and long short-term memory (LSTM) neural networks (on a basis of spatial rather than temporal relationships). However, the original choice of a *k*-NN algorithm appeared to be the most successful (as measured by accuracy of classification), as well as one of the simpler and quicker methods. Similarly, a neural network with numeric feature input performed most successfully overall as a second classifier.

**Figure 3:**
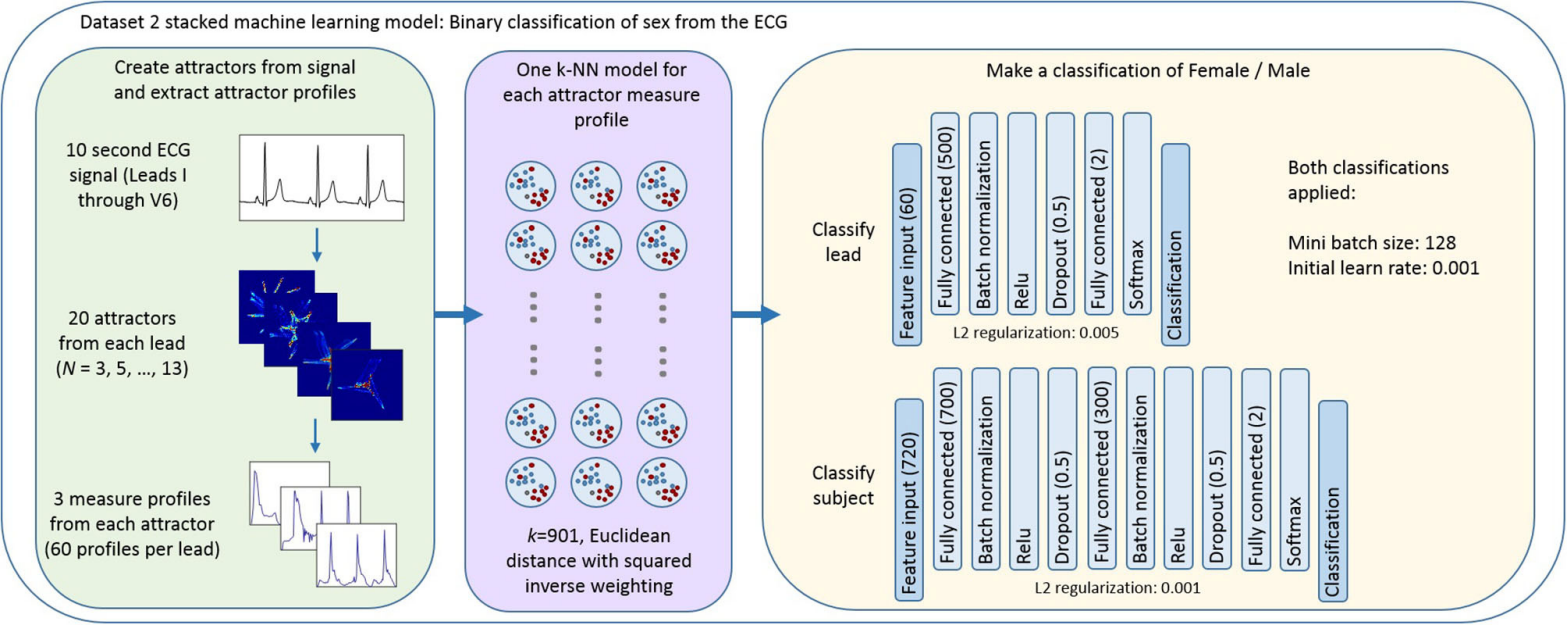
Overview of the stacked machine learning model. Each 10 second ECG lead was used to generate 20 N-point attractors (*N* = 3, 5, …, 13), each of which is quantified by three measure set profiles. A k-NN algorithm provides a classification of sex for each measure set (r density, θ density, attractor outline r) for each attractor. The posterior probability scores of each of these classifications formed the feature input to a neural network classifier which provided a classification of sex at (i) a lead level; and (ii) a subject level using data from all the 12 leads. Figure 4 provides the ranges of parameters considered when optimizing the model and further detail of the decisions made in defining the machine learning process and the cross validation approach is provided in the Supplementary Materials.

**Figure 4:**
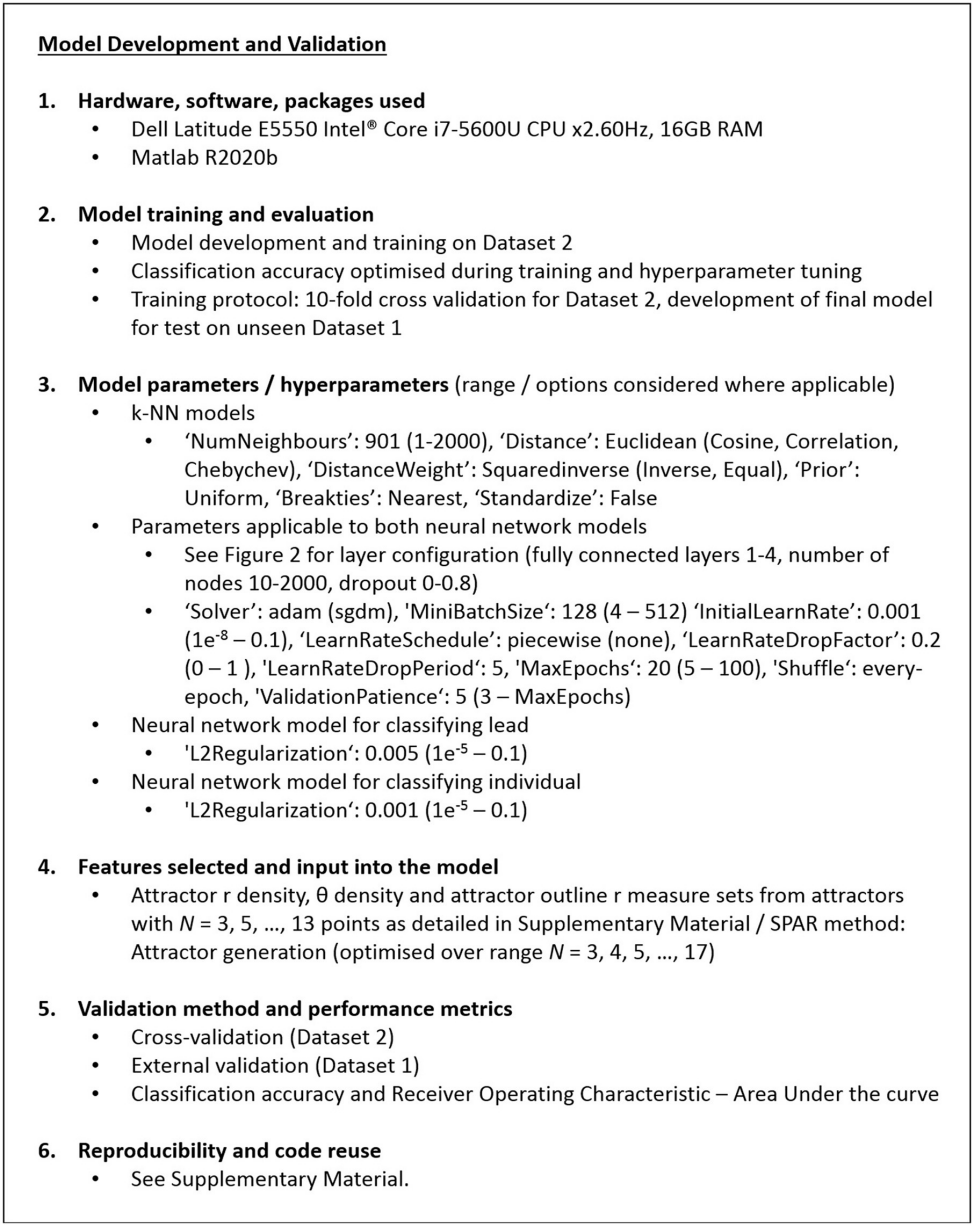
Detail of the machine learning model development and validation. The machine learning model parameters and hyperparameters are set to those given, with the values in brackets being the ranges/options considered during optimization of the model.

Thus the first step of our final model was the application of a *k*-nearest neighbors (*k*-NN) algorithm to provide a classification of sex for each measure set (r density, θ density, attractor outline r) for each attractor. We then applied a stacked approach (28), taking the posterior probability scores from the measure set classifiers as the feature input to a neural network to perform a classification of sex at a lead level and at a subject level using data from all the 12 leads. Figure 3 provides an overview of this process with further details about the *k*-NN and neural network parameters. The parameters and hyperparameters of the final model were determined by prior experience of our work in this space, grid searches and Bayesian optimization techniques. Figure 4 provides the final parameters selected and the range of options considered in the optimization process.

A detailed explanation (29) of the decisions made in defining the machine learning process is provided in the Supplementary Material.

### Application of the Machine Learning Model

As described above, we applied ten-fold cross validation within Dataset 2, and reported the cross-validated accuracy. A master model was then generated using all the data in Dataset 2 (*n* = 8,904). Dataset 1 (*n* = 104) was then used as an unseen test set on this final model.

We chose to present our classification results by accuracy and receiver operating characteristic (ROC) area under the curve (AUC) to allow comparison with other recent results in this space (17, 18). Whilst simple accuracy is not an appropriate metric when the prevalence of classes differs significantly, it is suitable and easily understood in the case of sex.

## Results

The first part of our study investigated the smaller, well characterized Dataset 1, and identified key attractor difference between sexes. We then determined SPAR's accuracy in discriminating between two biologically distinct groups in Dataset 2, which allowed us to demonstrate the application of SPAR with a much larger sample size.

### Attractor Appearance for Typical Female and Male Subjects From Dataset 1

In many cases, visual differences could be seen between female and male attractors for a given lead. Figures 2A,B shows the typical appearance of a female and male 3-point attractor from lead II signals. Whilst subtle differences can be seen in the two original signals, particularly in the ST segment, they would be difficult to quantify directly from the signal. However, the attractor amplifies small changes, making them easier to visualize and quantify. The overall appearance of the female attractor is one of a more dispersed distribution with wider arms, whereas the male attractor shows higher density, narrow arms. More subtle aspects of the signals are also reflected and these are more readily observed in differences between the measure profile plots, as shown in Figure 2C.

It is also noticeable on Figures 2A,B that the upper arm of both attractors is more variable than the other two arms. For both subjects, this is the result of the beat-to-beat variation in the T to P (interbeat) interval.

### Visualizing Attractor Differences by Sex Across All Dataset 1 Subjects

Although the primary use of the measure set profiles is to quantify an individual attractor image for an individual subject, they can be extended to visualize the profiles for a number of subjects together. This complementary presentation allows us to identify potential similarities and differences in the attractors of a population.

**Figure 5:**
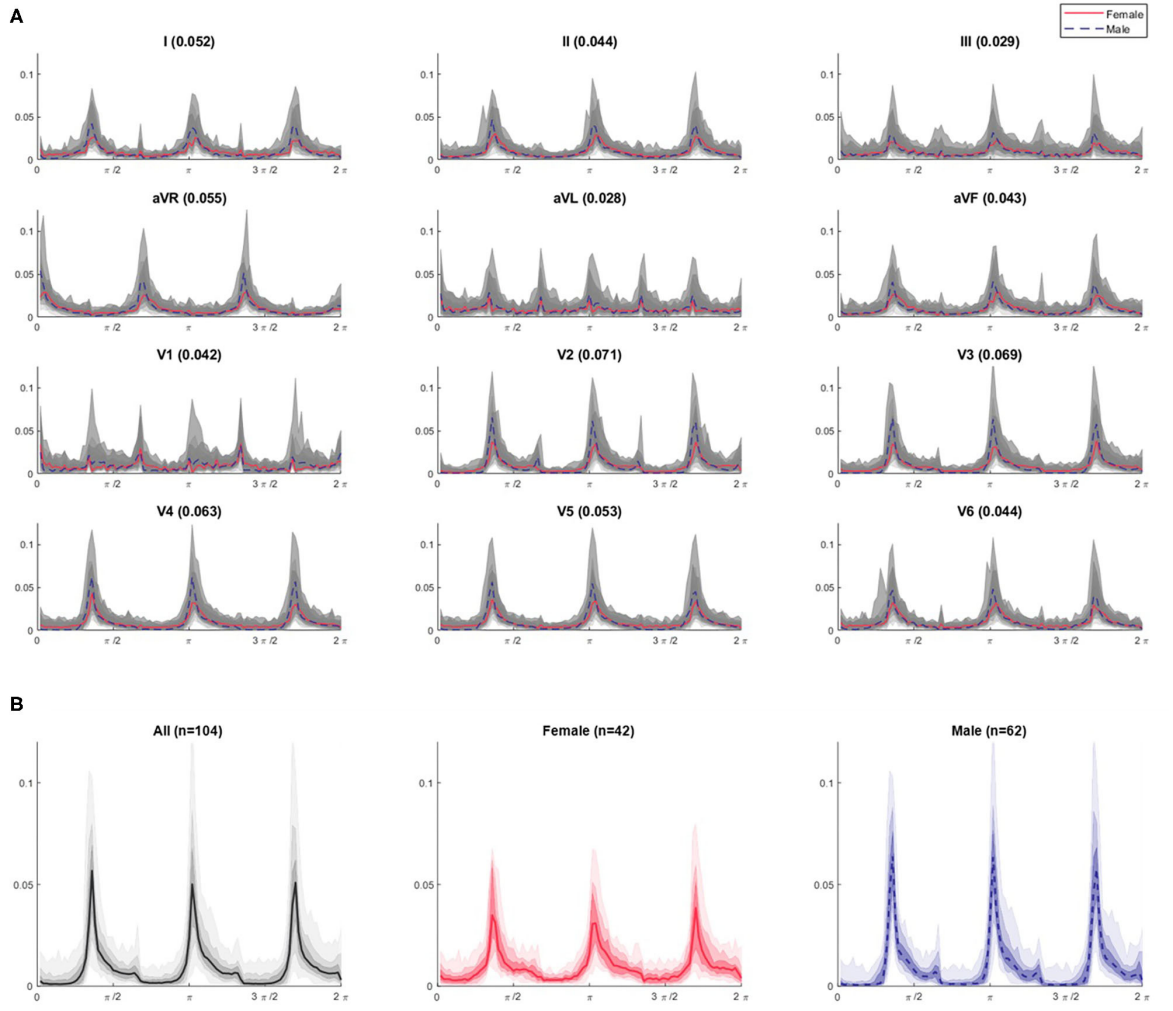
θ density profiles from 3-point attractors by lead for Dataset 1 **(A)** with a focus on the lead V3 profiles **(B)**. **(A)** The appearance of the profile over all subjects is shown in gray and appears relatively homogeneous for a given lead. The shaded gray bands provide the centiles on data, with the darkest being 25th – 75th centile, extending to all the data with the lightest band. However, when the median profile is split between female (red solid line) and male (blue dotted line) subjects, differences between the sexes can be seen. A simple quantification of the difference can be provided by considering the Euclidean distance between the female and male median lines, and is given by the bracketed number in each plot title. These distance values are greatest for leads V2 through V4 (0.071, 0.069, 0.063, respectively), supporting our visual interpretation of the plots as being where the largest sex differences occur. Similar observations can be made on the r, θ and attractor outline r profiles for the other N-point attractors, especially regarding differences in the mid-precordial leads. **(B)** For lead V3, we can clearly observe different appearances if we separate the profiles by sex. Female attractors appear to have lower θ density peaks and a more general spread of density across the attractor, compared with the higher θ density peaks of males contrasting with areas of very low density.

In illustration of this, Figure 5A shows the combined θ density profile plots of the 104 Dataset 1 subjects across all 12 leads. The appearance of the profile as shown in gray is relatively homogeneous within a given lead. However, when the median profile is split between female and male subjects, clear differences between the sexes can be seen. A simple quantification of the difference can be provided by considering the Euclidean distance between the female and male median plot lines. These distance values, as shown in Figure 5A, are greatest for leads V2 through V4, supporting our visual interpretation of the plots as being where the largest sex differences occur. Similar observations can be made on the r, θ and attractor outline r profiles for the other *N*-point attractors, especially regarding differences in the mid-precordial leads.

Figure 5B shows the 3-point attractor θ density profile for lead V3 in more detail. Whilst the 104 subjects of Dataset 1 present a reasonably homogeneous appearance of θ density in Figure 5A, separating this by sex, showed that the female attractors appear to have lower θ density peaks and a more general spread of density across the attractor, compared with the higher θ density peaks of males contrasting with areas of very low density.

**Figure 6:**
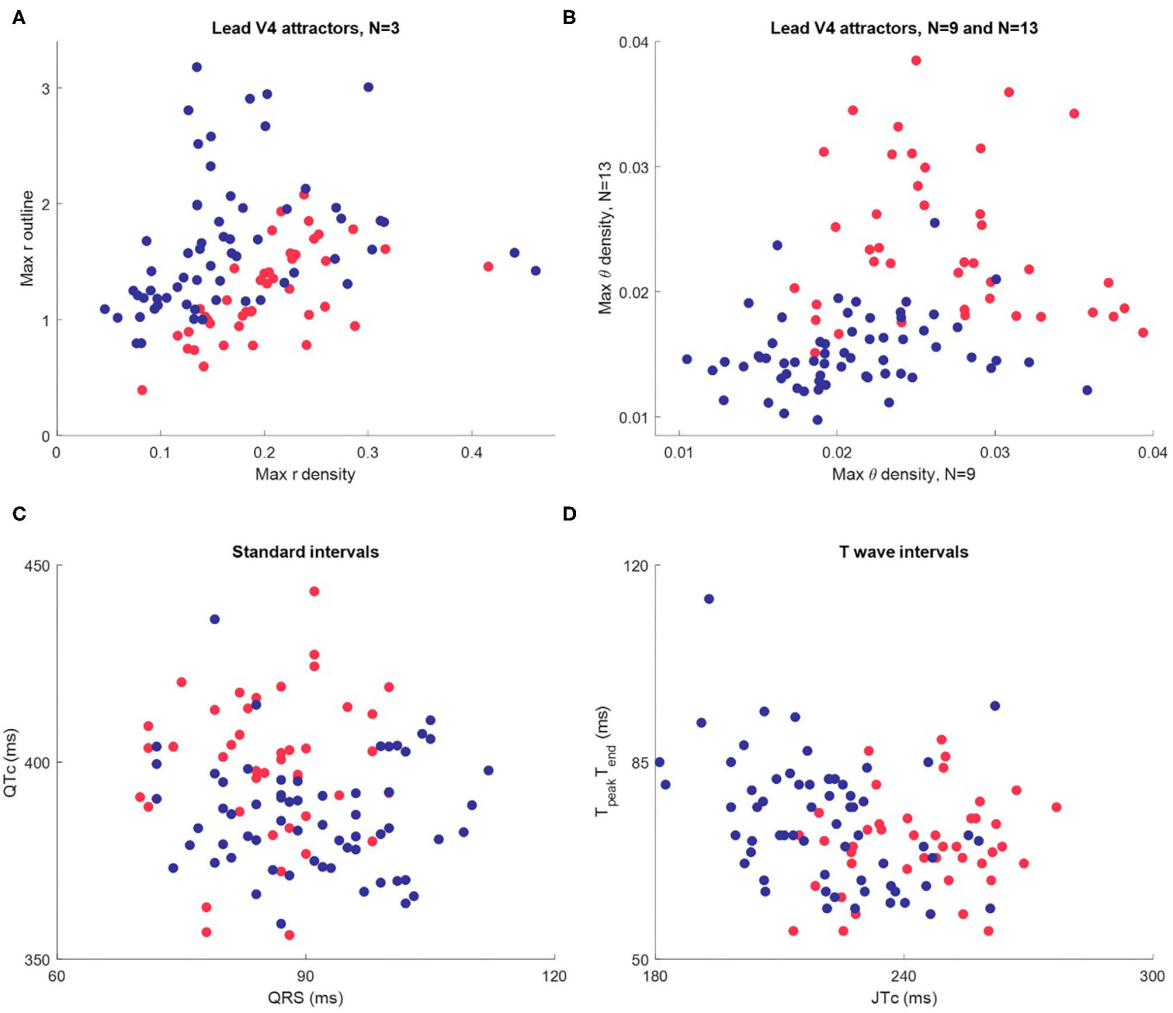
Scatterplots of maximum attractor measures and interval measures for Dataset 1 (*n* = 104, female in red, male in blue.) **(A)** The maximum value of each the r density and attractor outline profiles of lead V4 for 3-point attractors. **(B)** The maximum value of the θ density profiles of lead V4 for a 9-point and a 13-point attractor. **(C)** Standard intervals QTc and QRS. **(D)** Nuanced interval metrics JT_peak_c and T_peak_T_end_ which segment the T wave. Whilst the interval metric scatterplots indicate differences between the sexes, the visual distinction is not as clear as in the scatterplots using SPAR metrics.

A final visualization of the method in this part of the study involved taking only the maximum value of each measure profile for each subject. This gave three basic metrics, one from each of the r density, θ density and attractor outline r measures of the attractor image. Scatterplots using these just these basic metrics as shown in Figures 6A,B indicated a reasonably clear separation by sex and suggested that all three metrics (r, θ and outline r) and multiple *N*-attractors contributed to this distinction. Although the simple plots in Figures 6A,B utilized just three metrics (the maximum values) from the measure profiles, the shape of the whole profile (as in Figure 5) conveys more nuanced information, so we used the complete profile in our subsequent analysis.

Whilst the primary purpose of this study was not a direct comparison with interval measures, we did review these in Dataset 1, since well validated metrics were provided. Differences in certain interval measures between the sexes have been well documented (16, 30). We therefore briefly considered how they compared to our visual attractor analysis of the ECG. Additional details about the determination and distribution of interval measures in Dataset 1 can be found in the Supplementary Material.

A scatterplot of the standard intervals QTc (by Friderica's correction) against QRS is shown in Figure 6C. Whilst the plot suggested that many females have a higher QTc and lower QRS (as we would expect), the visual distinction between the sexes was not as clear as in the scatterplots of SPAR metrics. We then considered more nuanced T wave metrics, JT_peak_c (where JT_peak_c = JT_peak_ /RR^0.58^ with RR in seconds) and T_peak_T_end_, as shown in Figure 6D. We observed that females tended to have a higher JT_peak_c, but, once again, the visual distinction between the sexes was less clear than in the SPAR metric scatterplots.

### Dataset 2: Classification of Sex Differences in a Larger Population

**Figure 7:**
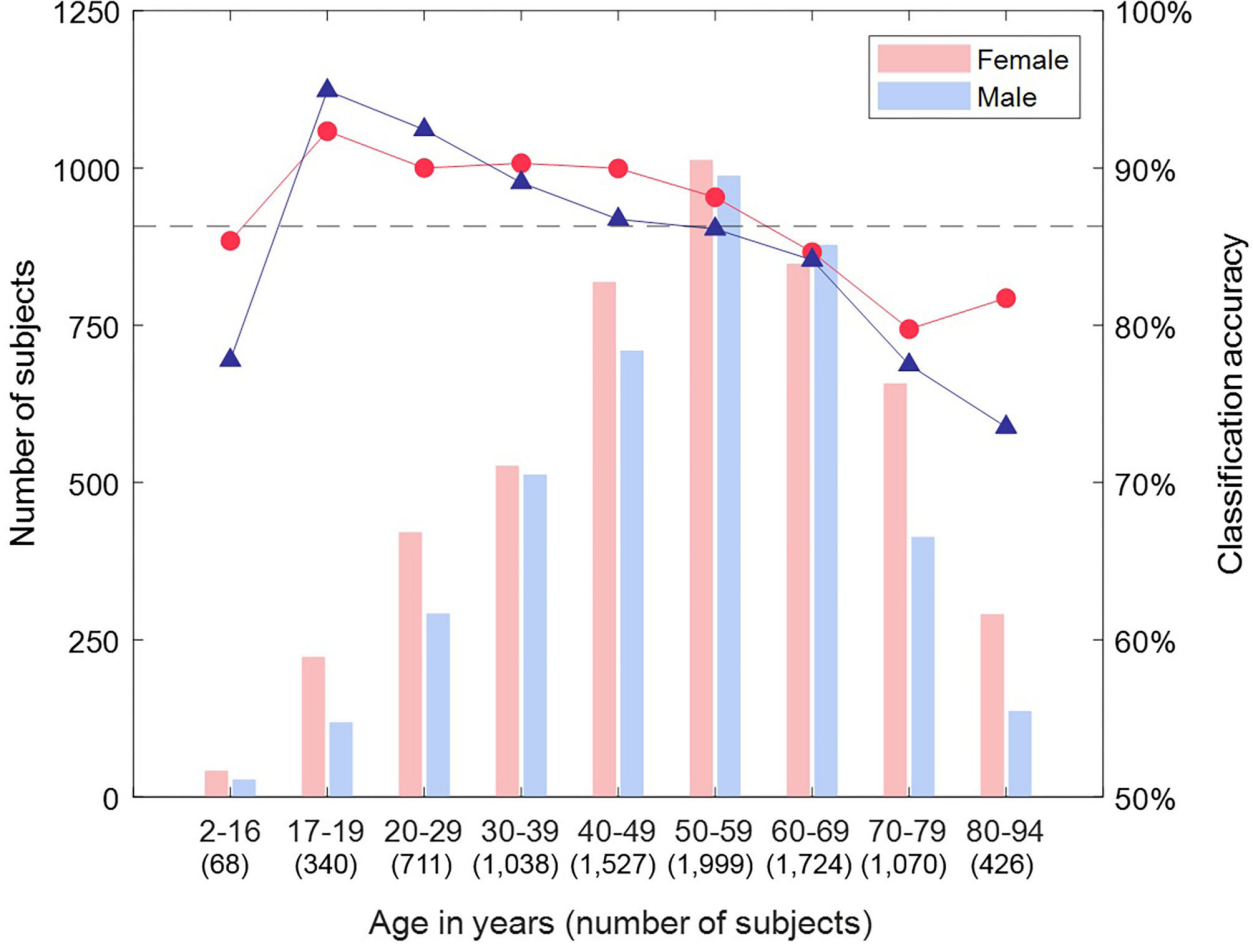
Classification accuracy by sex and age group for Dataset 2. Breaking down the classification result by age showed that accuracy is highest for young adults through to 60 years old. In particular, young adult males were classified most accurately. The accuracy for children and older adults is lower, particularly falling for males over 70 years.

We then tested the attractor's ability to discriminate between female and male ECGs in Dataset 2 (8,903 subjects.) Classification accuracies by lead and subject are shown in Table 1. The cross-validated classification model achieved a classification accuracy of 86.3% (AUC 0.93), with a sensitivity of 86.9% and specificity of 85.5% (with respect to the female sex.) The classification accuracy differed across the leads of the ECG, with the mid-precordial leads providing the highest accuracy. The overall classification of a subject exceeded any result from a single lead, indicating that information from multiple leads was required, and we found that the best classification was achieved from all 12 leads. Breaking down the classification result by age, as illustrated in Figure 7, showed that accuracy is highest for young adults through to 60 years old. In particular, young adult males were classified most accurately. The accuracy for children and older adults is lower, particularly falling for males over 70 years.

**Table 1:** Accuracy of classification of sex by lead and subject.

**Lead**	**Dataset 2** (***n*** **=** **8,903)** **Cross-validation**		**Dataset 1** (***n*** **=** **104)** **Unseen test**	
	**Acc**	**AUC**	**Acc**	**AUC**
I	70.8%	0.78	76.9%	0.86
II	69.3%	0.76	80.8%	0.87
III	61.5%	0.65	78.8%	0.84
aVR	71.0%	0.78	77.9%	0.90
aVL	66.4%	0.72	66.3%	0.72
aVF	65.5%	0.71	74.0%	0.82
V1	70.8%	0.78	74.0%	0.84
V2	77.0%	0.85	89.4%	0.94
V3	79.1%	0.87	79.8%	0.92
V4	79.8%	0.88	80.8%	0.91
V5	76.3%	0.83	77.9%	0.86
V6	73.4%	0.80	77.9%	0.84
**Subject**	**86.3%**	**0.93**	**91.3%**	**0.97**

*Classification accuracy and AUC value for the cross-validation (Dataset 2) and the unseen test (Dataset 1) on the final model generated from Dataset 2. The classification accuracy differed across the leads of the ECG, with the mid-precordial leads providing the highest accuracy. The overall classification of a subject exceeded any result from a single lead, indicating that information from multiple leads was required, and the best classification was achieved from all 12 leads*.

### Dataset 2: Machine Learning Classification Score and Confidence in the Classification

**Figure 8:**
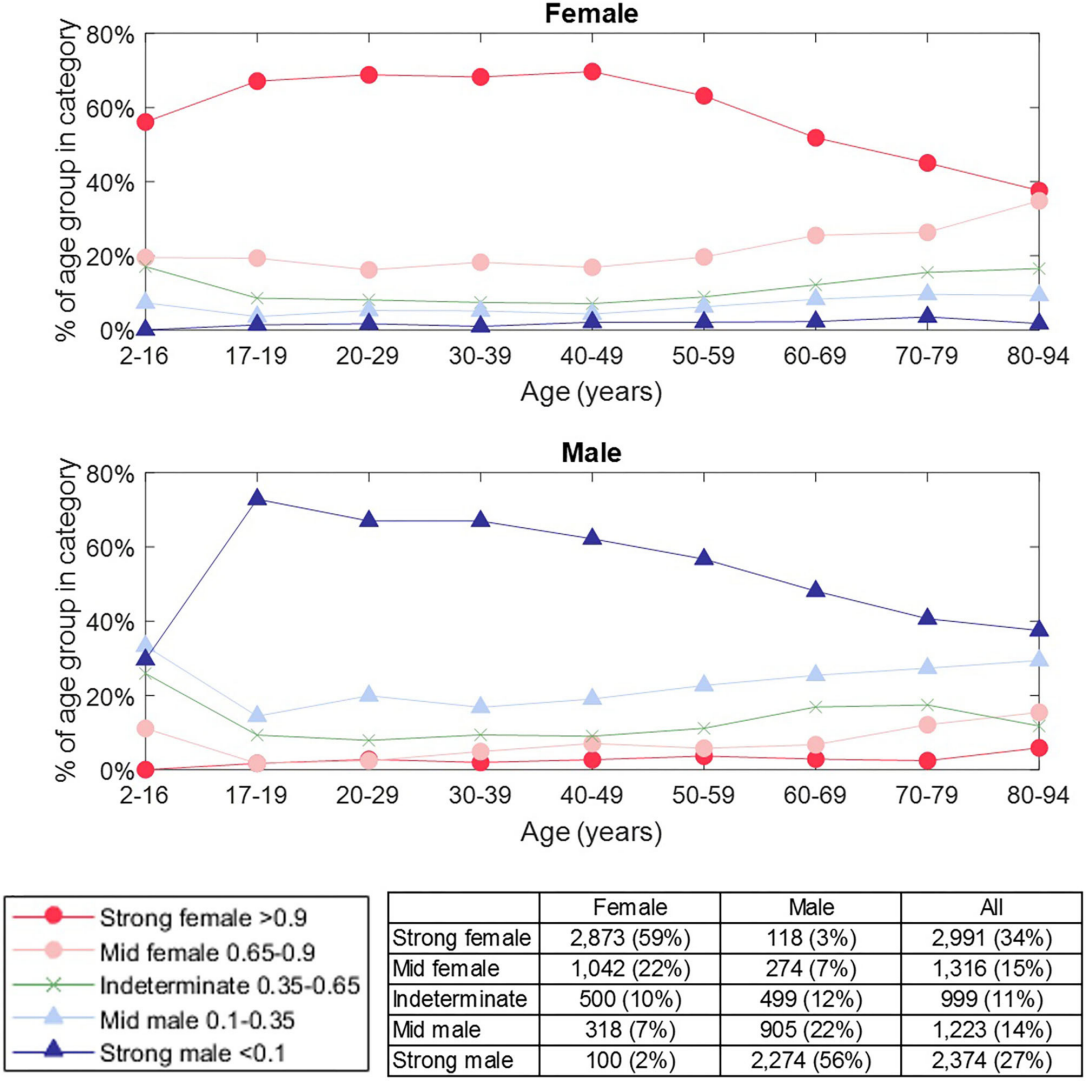
Prevalence of classification category by sex and age group. The classification category is defined by the classification score for a subject, as indicated in the legend. The number of female / male subjects in each category is shown in the table. The proportion of “strong male” classifications peaked in young adults and then declined, whereas the proportion of “strong female” classification did not begin to decline until around 50 years. Subjects were more likely to be classified as “indeterminate” than to have a “mid” or “strong” score of the opposite sex (other than for the oldest male category), which supported that the score can be related to our confidence in classification.

Whilst our results give a binary classification of sex, the basis of this is a score of between 0 and 1 (<0.5 male, >0.5 female) for each subject. We considered whether this score could indicate confidence in the classification outcome and defined the categories: strong female (score >0.9), mid female (0.65-0.9), indeterminate (0.35–0.65), mid male (0.1 to 0.35), strong male (<0.1). Figure 8 shows how the results are distributed across these categories by age. There were 60.3% of subjects that were classified as either “strongly male” or “strongly female”, and, within this group, the accuracy of classification was 95.9%. Furthermore, subjects were more likely to be classified as ‘indeterminate’ than to have a “mid” or “strong” score of the opposite sex (other than for the oldest male category), which supported that the score can be related to confidence in the classification. We also observed that the proportion of “strong male” classifications peaked in young adults and then declined, whereas the proportion of “strong female” classifications did not begin to decline until around 50 years.

**Figure 9:**
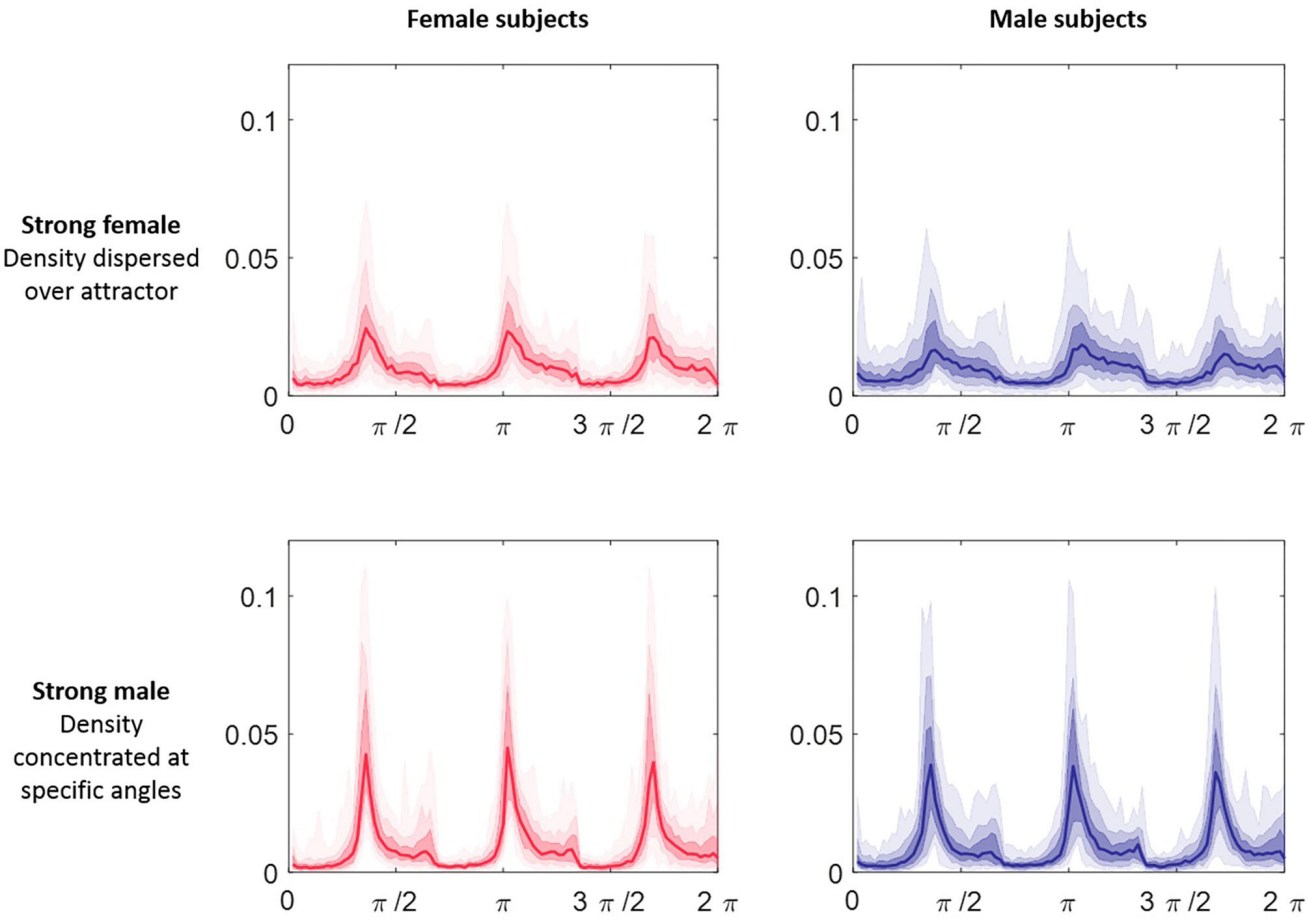
θ density profiles for a 3-point attractor from lead V3 split by sex and classification category. Each plot comprises 50 subjects selected at random from Dataset 2 in the categories of “strong male” and ‘strong female’ respectively. The median of the profiles is shown as the line plot, the first surrounding darker band is the 25th to 75th centiles and then extending out with further bands to cover all the data. The appearance of the profile for the two categories was very different; the “strong male” profile showed sharp density peaks, whereas the “strong female” indicated a more even density distribution across the attractor. We observed that the greatest visual similarity was between subjects of the same category, irrespective of their actual sex.

The attractor measure profile plots provided further insight into our categories of classification confidence. We selected the attractors of 50 females and 50 males randomly from each of the categories (strong female, mid female, indeterminate, mid male, strong male). On reviewing the measure profile plots for each category, we observed that the greatest visual similarity was between subjects of the same confidence category, irrespective of their actual sex. Figure 9 shows the θ density profile for a 3-point attractor on lead V3 split into “strong female” and “strong male” categories. The appearance of the profile for these two categories was very different; the “strong male” profile showed sharp density peaks, whereas the “strong female” indicated a more even density distribution across the attractor. Thus the profile for a category appeared consistent, and was independent of the sex of the underlying subjects.

### Testing New Data on the Machine Learning Model

**Figure 10:**
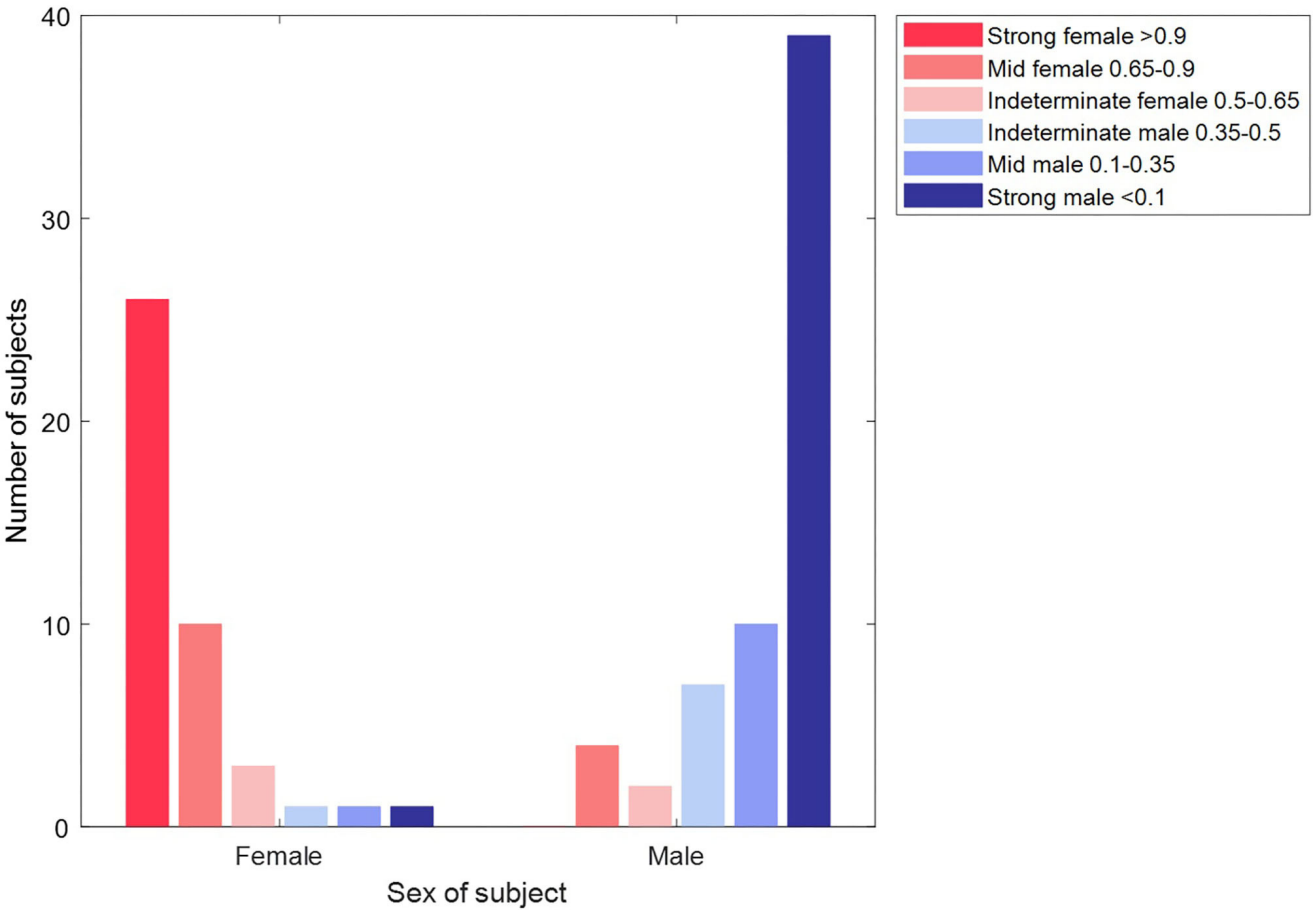
Classification of unseen records in Dataset 2 by sex and classification category. The plot shows the classification of each of the 104 subjects of Dataset 2. The majority of subjects (39/42 females in the red bars and 56/65 male subjects in the blue bars) are classified correctly. Furthermore, the majority (65/95) of these correct classifications are made with a “strong” confidence.

As a concluding test of our classification, we created a master machine learning model from all the Dataset 2 data and took the 104 subjects of Dataset 1 as an unseen test set. From the age profile of these subjects, our cross-validation indicated an expected accuracy of 89.6%. The actual classification slightly improved on this with an accuracy of 91.3% (AUC 0.97). Figure 10 shows how each subject was classified across the strong female, mid female, indeterminate, mid male and strong male categories defined above. Nine records (three female) were classified incorrectly. Whilst we observe that incorrectly classified records present attractors that are consistent with the attractor profiles for their category (so the machine learning classification is as we would expect), the number of records involved is too low to comment further on specific characteristics of these subjects.

## Discussion

Our primary motivation was to demonstrate the accuracy of the SPAR method in discriminating between two biologically distinct groups. In this proof-of-concept study, we have successfully demonstrated the utility of SPAR in distinguishing sex differences from normal sinus rhythm ECGs. Whilst sex was used as an arbitrary classifier, our results support the wider clinical value of SPAR and also highlight the importance of acknowledging sex differences during clinical ECG interpretation.

The SPAR method takes a holistic approach to signal analysis, and provides an innovative visual representation by transforming an approximately periodic waveform into a two-dimensional image called an ‘attractor’. Utilizing the whole ECG signal in this way would make better use of data that is already available and would not require a change in signal acquisition protocol. Importantly, we recognize that to support clinical uptake, a method must be intuitive, time efficient and not necessitate complex pre-processing which can introduce bias. As SPAR has been developed to provide a visual image of signal morphology and can be generated in real time on the output from existing devices, we believe that SPAR addresses these common issues which may otherwise lead to resistance in clinical implementation.

The ECG signals used in this study were obtained from publicly available data and no further pre-processing was applied to them. Whilst ECG signals are susceptible to corruption by noise and other artifacts, the 10 second ECG recordings we applied here had been considered sufficient for research or diagnostic purposes (as dependent on the database), supporting our decision to not filter or otherwise process them additionally. Furthermore, the SPAR method has been shown to act as a high-pass filter to the lowest frequencies (8) (also, Lyle JV, Aston PJ, submitted) and so baseline wander is naturally attenuated by the method. It is beyond the scope of this study to make a formal assessment of the impact of noise on classification since the data applied here has been determined to be adequate clinically. However, we have utilized the SPAR method in a consideration of noise robustness in deep learning (31) and look to extend this in future studies. In the real-world application of the SPAR method, existing signal quality algorithms (32) can be applied to longer signals to extract sufficiently clean data, as would be undertaken routinely for the extraction of diagnostic ECG from such signals.

We observed that the SPAR attractor amplified subtle details from the ECG waveform, and we saw clear visual differences between female and male ECG signals. Attractors from Dataset 1 subjects (*n* = 104) were visualized together using the measure profile plots. Many of the differences observed in these plots could be explained by sex when considered at a group level. We extended this basic analysis with machine learning techniques to successfully classify subjects by sex from the much larger Dataset 2 (*n* = 8,903), and found that interpretation through the attractor measure profiles provided visual insight into these results.

Within our review of Dataset 1, we observed that although the standard interval and more specific repolarization metrics (JT_peak_c, T_peak_T_end_) presented some visual difference by sex, these differences were more pronounced in measures drawn from the attractor. Furthermore, using the attractor has an operational advantage over interval measures. Beyond a determination of the RR interval [for which numerous robust algorithms are available (33, 34)], an accurate detection of other fiducial points, such as the J point and end of the T wave, remains compromised (35). Conversely, the SPAR method is motivated by the qualitative assessment that a clinician will make on the morphology of the whole waveform, information that is discarded when only intervals and amplitudes are assessed. The SPAR method removes the need to make assumptions about a signal. It can be considered an averaging of the cycle characteristics before quantifying them, which makes it more robust when signals are noisy and limits the introduction of bias arising from manual intervention.

The QT interval is known to show a significant difference between the sexes, with the ST segment and start of the T wave providing the biggest contribution to the longer QT interval observed in females (36). In support of this, we consistently made observations during this study that indicated the sex differences we saw in the attractor were related to aspects of the repolarization features of the ECG. The major 3-point attractor differences of wider arms observed in Figure 2 resulted from a higher QT:RR ratio in the female signal. Furthermore, the greatest sex differences in the measure profile plots for multiple subjects and the highest individual lead machine learning classification accuracies were seen in the mid-precordial leads, which typically present the highest amplitude T waves in an adult ECG recording (37).

Whilst the mid-precordial leads provided the highest individual lead accuracy, we found that the best overall machine learning classification accuracy was obtained using all 12 leads of the ECG, which suggested that subtle differences in the overall pattern of presentation contributed to the classification and that features from across the whole waveform were involved. This finding is supported by two recent deep learning classifications of sex in the ECG (17, 18), both of which reported results based on the input of a 12-lead ECG, rather than separate leads. Additionally, Attia et al. also considered a classification on separate parts of the waveform, which gave an inferior result to utilizing the whole signal (17).

The need for the supplementary information from all 12 leads for the most successful classification suggests that single lead recordings may have some limitations when assessing the ECG waveform. In our future work on the SPAR method, we will focus on its application to single lead ECGs in support of patient stratification and indication for referral in primary care. We have previously had some success working with just lead II signals in subjects under pharmacological challenge (24) and look to extend this.

The machine learning cross-validation accuracy we reported in this study approached the classification accuracy of recent much larger studies (17, 18), and our unseen test accuracy compared favorably with these results. We focused on non-pathological ECG presentation to allow us to understand more about the attractor representation of the signal without confounding disease states, but this constrained our data size when compared with the larger studies, and we aim to extend our classification to broader ECG presentations. Our classification accuracy may have been limited by the relatively small number of records in the child and over 70 age groups, and the greater number of females in these groups. However, we achieved a promising result with around 1% of the data used by Attia *et al*, which supports the use of the attractor for work on smaller datasets where a full deep learning solution would not be applicable due to an insufficient quantity of data.

Our cross-validation and unseen test results showed good agreement with the literature on classification accuracy by age group. Whilst we had only a small number of child ECGs, we classified them with an accuracy of 82.4%, although we were more successful with female classification than male. Most observable sex differences are believed to occur from adolescence, and the difference in the QT interval is thought to result from a shortening of the interval in males (38), supporting that we would be more likely to misclassify male children as female. The most clear sex differences in the ECG are observable in younger adults and tend to decline from 50 years (7, 39), and we saw a similar pattern to this with our highest classification accuracy in young adults and a decline of accuracy as age increased.

A key aspect of the attractor is its independence from the heart rate, since the spacing of the points used to generate the attractor is determined from each signal's cycle length. Although healthy subjects demonstrate a higher mean resting heart rate in females than males (40), including this additional information with the attractor measures in the machine learning model resulted in a slightly lower sex classification accuracy. As the heart rate has a complex non-linear effect on the ECG morphology (35, 41) and the attractor reflects the morphology of the waveform, this may suggest that the sex differences observed in heart rate are captured implicitly in the attractor and that more subtle aspects of the signal are more useful for the classification of sex than the explicit heart rate.

The classification scores from our machine learning model were used to further categorize our binary classification of sex. Our observations on the “strong female” and “strong male” category for subjects aged 17–50 years (see Figure 8) had similarity to the prevalence of sex-specific patterns of repolarization identified by Surawicz and Parikh through a manual review of leads V1-V4 (42), with the ‘strong male’ declining in prevalence, whilst the “strong female” category prevalence remains more consistent. For older adults, we observed that our “strong” categories decline whilst “mid” categories become more prevalent. However, we did not observe the steep drop in male pattern prevalence seen by Surawicz and Parikh. One explanation for this is that the specific features focused on by Surawicz and Parikh (the J point and the T upslope angle) are significant for determining sex differences in younger adults, but that differences in other parts of the signal have importance in older adults (especially male) and the SPAR method quantifies these by capturing the whole waveform and not just the repolarization.

Our findings using the score categories indicated that a simple binary classification is not always sufficient to capture the subtleties of a subject's ECG. By using the attractor measure profiles to visualize multiple attractors together, we saw that for some subjects, the appearance of their attractor may be more closely matched to a typical one of the opposite sex (see Figure 9). For example, we observe that male ECGs that were classified incorrectly as “strongly female” have an attractor that is consistent with those of female ECGs (and vice versa, whereby incorrectly classified female ECGs have attractors that visually resemble male ECG attractors). In terms of the classification problem, this observation suggests that misclassification of a subject's sex may be due to this biological effect rather than failure of the machine learning algorithms, and so a significant improvement in classification performance may not be possible. The consistency between our results and those of recent studies (17, 18) indicates that the limit of performance for this simple binary problem may have been reached and that different algorithms would perform similarly. In terms of future work, this natural clustering of the data to attractor appearance rather than a subject's sex suggests that a more refined, personalized stratification based on a pattern of presentation is more appropriate rather than just considering an individual's sex alone.

## Conclusion

In this study, we used the recently developed SPAR method to investigate the non-pathological ECG signal in the context of sex differences. The SPAR approach uses the whole waveform, rather than drawing individual features from different parts of the ECG, and this allowed us to discriminate sex differences. Our observations of how these differences presented in the attractor corresponded to the existing literature for characteristics of female and male ECG signals. However, using the attractor provides a simple and robust means of quantifying the morphology underlying these differences.

The extension of our work into a machine learning classification of sex demonstrated that the SPAR method could be successfully applied across a larger dataset. Furthermore, the attractor profiles can be used as a visual tool to provide an understanding of why a specific classification was given. Whilst this does not wholly overcome the “black box” nature of machine learning techniques, it can provide complementary information.

Prior studies classifying sex from the ECG present a classification only using all 12 leads combined. We considered the classification for each lead separately and in differing combinations, but concluded that the best classification was achieved using all 12 leads. Although this was in the context of the specific problem of sex classification, it indicates that single lead recordings may have some limitations when assessing the ECG waveform and that this should be considered in future work.

We appreciate that the simple classification of sex is not directly a clinical question, and nor would it be practical to limit the application of the SPAR method to non-pathological signals only. However, this proof-of-concept study showed that subtle differences in the ECG can be amplified by the attractor. The detection and quantification of more nuanced ECG differences in this way could support management of clinically significant issues, such as patient stratification for drug induced risk of arrhythmic events (43, 44). Our findings also emphasize the importance of considering sex and age differences in determining reference ranges and clinical management protocols.

We acknowledge that morphological ECG appraisal is already undertaken by experienced clinicians and electrophysiologists, and introduce SPAR as a means of automating this process. Although the attractor provides a unique visualization of a signal, it is not necessary for a clinician to learn to interpret the image itself. We can apply various techniques, such as the measure profile plots and machine learning, which could be coupled to an alert or simple metric, to present a clinically meaningful output to the end user. For example, in this study, we utilized the classification score to provide a measure of confidence in our output. In a clinical setting, this approach would allow for greater manual effort to be directed at those results that are deemed ‘indeterminate’. Additionally, SPAR could be used to supplement conventional fiducial point analysis, and may enhance the sensitivity of identifying patients requiring referral or review.

The SPAR method was developed in response to the challenge of making better use of high fidelity physiological waveform data that is already recorded, and its simple implementation overcomes many of the traditional barriers to clinical uptake. Our successful discrimination of sex by SPAR demonstrated the accuracy of the method in two biological distinct groups and supports the further development of SPAR in the areas of patient stratification and risk management.

## Data Availability Statement

The datasets generated for this study can be found in online repositories. The names of the repository/repositories and accession number(s) can be found in the article/Supplementary Material.

## Ethics Statement

All data used is open access on PhysioNet.

## Author Contributions

JL, MN, and PA contributed to conception and design of the study. JL obtained the data and executed the study, with regular input from MN and PA in the discussion of results. JL wrote the first draft of the manuscript. All authors contributed to manuscript revision and approved the submitted version.

## Funding

JL was supported in this work by an EPSRC Ph.D. studentship (EP/MQ15508160/1).

## Conflict of Interest

PA and MN have a patent WO2015121679A1 “Delay coordinate analysis of periodic data”, which covers the foundations of the SPAR method used in this paper. The remaining author declares that the research was conducted in the absence of any commercial or financial relationships that could be construed as a potential conflict of interest.

## Publisher's Note

All claims expressed in this article are solely those of the authors and do not necessarily represent those of their affiliated organizations, or those of the publisher, the editors and the reviewers. Any product that may be evaluated in this article, or claim that may be made by its manufacturer, is not guaranteed or endorsed by the publisher.
