# Supplementary material for: Symmetric Projection Attractor Reconstruction: Sex Differences in the ECG
Source: Front Cardiovasc Med. 2021 Sep 23;8:709457. doi: 10.3389/fcvm.2021.709457 (PMC8495026; doi:10.3389/fcvm.2021.709457)
Supplement: Supplementary file 1 [file Data_Sheet_1.zip › Supplementary Material for Symmetric Projection Attractor Reconstruction Sex Differences in the ECG.pdf]

Supplementary Material

Further details are provided on all aspects of the Methods for the purpose of transparency and reproducibility.

DATA

Our justification for taking only one ECG signal per subject is that the highly individual nature of the ECG means that normal recordings for the same subject usually have a very similar appearance and, therefore, a very similar attractor. Since more than one recording was available for some subjects, the inclusion of all ECG signals could give skewed results. In taking only one recording, we then selected the first available ECG signal for each subject to apply a uniform methodology. This approach was also taken in the deep learning study on sex classification by Attia *et al* (1) for the same reasons.

The Physionet (2) databases used to create Dataset 1 and Dataset 2 are accompanied by comprehensive documentation, as referenced within the main paper and below. Supplement Table 1 summarizes the key characteristics of each dataset split by sex.

Note on Dataset 1 interval metrics

The three studies (ecgrdvq, ecgdmmld and ecgcipa (3–6)) combined to create Dataset 1 included the standard interval metrics (RR, PR, QRS, QT) and more nuanced repolarization intervals (JT_{peak} and $T_{peak}T_{end}$). The determination of these intervals was first automated using information from the derived vector magnitude lead (applying data from across the 12 leads) with subsequent manual review by two ECG readers. Thus the interval metrics for this study will have a higher accuracy than those obtained by simple automation, although the methodology is time consuming and would not be practical for the analysis of large data volumes. We followed the justification of Vicente *et al* (6) in correcting the QT and JT_{peak} intervals for heart rate, taking Friderica's correction for QT ($QTc = QT/RR^{1/3}$ with RR in seconds) and a correction to JT_{peak}, ($JT_{peak}c = JT_{peak}/RR^{0.58}$ with RR in seconds), whilst making no correction in the $T_{peak}T_{end}$ interval, as prior studies suggest rate independence at resting heart rates.

Supplement Table 1 indicated that the interval measures of Dataset 1 are in agreement the expected differences between the sexes (7), including the lower RR, higher QTc and higher JT_{peak} in females. Supplement Figure 1 provides boxplots comparing the interval measures of Dataset 1 when grouped by sex. The Mann-Whitney U test was applied to compare the medians between the groups and significant differences (at the 5% level) are seen for RR, QTc and $JT_{peak}c$, as we would expect from the literature.

Note on Dataset 2 labels and stratification

The whole PTB-XL dataset comprises 21,837 clinical 10 second 12-lead ECG recordings from 18,885 patients captured between 1989 and 1996 (8). The recordings were assigned to one of five diagnostic superclasses: 'Norm', 'Myocardial Infarction', 'ST/T Change', 'Conduction Disturbance', 'Hypertrophy'. 73.7% of all recordings were marked as having human validation of the diagnosis, whilst the remainder had only interpretation by the ECG device.

We took all first recording with the label 'Norm' for each subject. Of these, 83.6% had been validated by a human, which suggested that interpretation by ECG device may be more likely to provide an abnormal label

incorrectly (for example in the presence of noise) and provided confidence that those labelled 'Norm' are predominantly correct.

We also note that we provided our own random stratification folds for the cross-validation on Dataset 2 and include these in an additional supplementary file (see ADDITIONAL ACCOMPANYING FILES in this Supplementary Material). Although suggested machine learning folds are provided with the PTB-XL metadata, these did not provide numerically balanced folds (or a balance by age and sex) when we selected for the first 'Norm' recordings only.

SPAR METHOD

Attractor generation

The SPAR technique to generate a two-dimensional image (an 'attractor') from an approximately periodic signal is well documented (9) (also, Lyle JV, Aston PJ, submitted). Here we provide additional comments on the method applicable to this study. We emphasize that although the description of the attractor method may appear complicated, it uses existing data and is straightforward to implement, quick to run (can be run in real-time) and does not require manual intervention. Those unfamiliar with the SPAR method are encouraged to view Supplement Video I (see SUPPLEMENT VIDEO CAPTIONS in this Supplementary Material), which shows how a signal is transformed into a 3-point attractor.

The natural beat-to-beat variability in a real ECG signal means that the orbits in the two-dimensional attractor image will vary. Thus the attractor is presented as a density to indicate the areas that are traversed more or less frequently. Each orbit of the attractor corresponds to approximately one heartbeat, and so the attractor is explicitly independent of the RR interval of a given signal. However, differences in RR that lead to changes in ECG waveform morphology and natural variability between beats will be implicitly captured in the attractor.

In this analysis we utilized attractors generated with all odd numbers of points from $N = 3,\ldots,13$, which allows for different features of the waveform to be emphasized. Due to the symmetric properties of the attractor (9) (also, Lyle JV, Aston PJ, submitted), the use of even numbers of points results in overlapping of ECG features on the attractor and has proved to be less useful than the odd numbers of points in this case. For an odd numbers of points N, we generate $(N\text{-}1)/2$ two-dimensional projection attractors a_k, where $k = 1,\ldots, (N\text{-}1)/2$. Supplement Video II (see SUPPLEMENT VIDEO CAPTIONS in this Supplementary Material) shows the generation of the $k = 3$ projection attractors associated with using $N = 7$ points on the signal.

Where N and k have a common factor greater than 1, as in the case of $N = 9$ and $k = 3$, we find that the resulting attractor will have very similar characteristics to one generated from an embedding in a lower number of points (see Lyle JV, Aston PJ, submitted) and we exclude these from the analysis. Supplement Figure 2 shows all attractors generated from a subject's lead II signal for the numbers of points N applied in this study. We also note that Main Paper Figure 5(B) is generated taking attractors where $N = 9$, $k = 4$ and $N = 13$, $k = 6$, respectively.

Validation of the average cycle length and other pre-processing

The only pre-processing step required to generate the attractor is the determination of the average cycle length (ACL) of the signal, as this provides the fixed distance τ between the N points that create the embedding. We used a QRS detector based on the Pan-Tompkins method (10,11) to identify the R peaks on each 10 second lead I, lead II and lead V6 signal. The result from all three leads was utilized to provide a means of validating the determination of the ACL; for 96.3% of records, all three leads gave the same ACL within a small error

tolerance, and in a further 3.5% of records, two of the leads gave the same ACL. In Dataset 2, 18 (0.2%) subjects required review of their ACL and the correct cycle length was provided manually for these cases.

In Dataset 1, the ACL result was also compared with the validated RR interval provided in the dataset metadata. Interestingly, 12/104 values differed by 3 to 24 datapoints (noting that there are 1,000 datapoints per second in these signals, so these are small discrepancies.) These differences were all reviewed manually and arose due to R peaks occurring in close proximity to either the start or end (or both) of the 10 second signal. It appears that these peaks were excluded from the results obtained for the validated RR intervals in the metadata, whilst our QRS-based algorithm was returning the correct ACL for application in the attractor method which uses the whole of the signal waveform.

We did not perform any other pre-processing on these signals and did not exclude any signals that fell within our criteria for selection (all first recordings of all subjects in the three studies selected for Dataset 1, all first 'Norm' label recordings in Dataset 2.) Most of the signals were of good quality, and the attractor method implicitly introduces a filtering of very low frequencies (9) (also, Lyle JV, Aston PJ, submitted) which reduces the effect of baseline wander. Our approach to take the clinical signals as they are provided is similar to that applied to other studies with large data volumes (1,12), which allows comparability with these results and forms the basis for expansion to real world application where excessive selection and pre-processing is impractical.

Quantifying the attractor

An image on a plane can be quantified by considering its polar coordinates, the radial distance r and angular distance θ, and we take this approach to generate three sets of measures that summarize the ECG attractor. Each set of measures is illustrated in Supplement Figure 3 and we determine them as follows:

(i) the radial density distribution (r density) based on the distance from the centre to the outer edge of the attractor. We divide the distance from the centre to the outer edge of each attractor into 50 bins and simply sum up the number of points falling into each bin to give one distribution of the density of the attractor.

(ii) the θ density distribution (θ density). We apply a similar approach in the angular direction by sweeping around the attractor in a circular direction and dividing the 360 degrees traversed into 100 bins and again summing up the number of points in each bin to give an alternative distribution of the density of the attractor.

(iii) the outline shape of the image as the maximum distance r in the θ direction (attractor outline r). We combine the radial and angular measures by taking the maximum distance of the attractor from the centre for each of 100 angular bins.

The number of bins applied for the method was determined by optimization within the machine learning method and was based on considering a range of 20 to 250 bins.

MACHINE LEARNING

This text description of our machine learning model is supported by Main Paper Figure 3, Main Paper Figure 4 and Supplement Figure 4, which provide further detail on the design, development and implementation of the model following the recommendations of Stevens *et al* (13). In this supplementary section, we note that there is naturally some duplication of the content of the Main Paper so that this material can be read as an independently as a complete description of the machine learning methodology.

Machine learning was applied as a tool to help us recognize patterns related to the classification of sex in the larger data volume of Dataset 2. We have previously achieved a classification accuracy of sex of 96.3% in a preliminary study of a small dataset of young, healthy adults using a support vector machine algorithm (14).

More recently, Attia *et al* reported a 90.4% accuracy on the sex classification of 12-lead ECGs with a dataset of over 770,000 adults covering various disease states (1). Work by Strodthoff *et al* using the full PTB-XL dataset reported 84.9% accuracy (89.8% for subjects with a 'Norm' label) (12). In both of these studies, 10 second 12-lead ECG signals were taken as the input with no further pre-processing. However, the 'black box' nature of the deep learning techniques applied does not further our understanding of how these results are obtained or how they can be interpreted. Attia *et al* considered this by also performing a classification on some subjects using discrete portions of the ECG (such as the P wave and QRS complex), but the results compared poorly with using the whole signal, suggesting that features from the whole ECG waveform are important in the classification of sex differences.

Building on our previous work and the indication that the whole ECG signal is relevant in the classification of sex, it suggests using the attractor, which captures the morphology of all of the underlying waveform, as the basis of feature input to machine learning. The visual attractor output is a two-dimensional image, which would suggest the direct application of deep learning. However, our early studies on this approach (15,16) indicate that existing transfer learning is not particularly successful on attractor images and a much larger volume of images is required in the application of this technique. We therefore quantified the attractor image by the three sets of measures, r density, θ density and attractor outline r, and took these as our feature input to machine learning.

The early phases of our machine learning investigation for this study were exploratory to determine the most appropriate techniques for our features. We utilized our previous experience in machine learning on the attractor (14,16,17) and the clinical knowledge that the differences are likely to be subtle so we would be considering relatively weak predictors that build together to form patterns in the data. Thus we started by classifying our attractor measure sets with a *k*-nearest neighbors (*k*-NN) algorithm, which is also nonparametric, so we were not making any assumptions about how we were separating our data.

We investigated various structures to our initial model, from combining all measure sets in a single classification (either by lead or by subject) to considering separate classifications by measure set, number of points, projection *k* (based on attractors a_k) and lead. Our preliminary results indicated that the use of a stacked model (18) whereby the outcome of separate classifications were then combined in a second classifier for either the lead or the subject was the most successful (when measured by the accuracy of classification). We also determined that the use of the posterior probability scores of each separate classification provided a more useful input to the second classifier than a binary Female / Male label. On this basis, the second classifier is therefore combining a large number of weak predictors and our data volume indicated that a neural network with numeric feature input would be an appropriate classifier.

At different stages of the preliminary investigation, we also considered different machine learning algorithms as both the first or second classifier, including support vector machines (SVM), random forest and long short-term memory (LSTM) neural networks (on a basis of spatial rather than temporal relationships). However, the original choice of a *k*-NN algorithm appeared to be the most successful (as measured by accuracy of classification), as well as one of the simpler and quicker methods. Similarly, the neural network performed most successfully overall as the second classifier.

Within the framework, we considered the form of the feature input. The r density, θ density, attractor outline r measure sets are each determined as a distribution over a number of bins. We found that taking 50 bins for the r density and 100 bins for the θ density and attractor outline r measures (based on considering a range of 20 to 250 bins) was the most successful. Other features considered for input to the model were summary metrics of the r density, θ density and attractor outline r distributions (e.g. maximum, minimum and centile values) and the ACL (effectively the RR interval), but these were not as successful in classification.

We also considered different approaches to the stacked model in the determination of a classification for a subject. It was found to be less successful if we used a 'double stacked' model whereby the classification of each lead was then combined for a subject or if we used data from only a subset of the 12 leads. This latter point is of particular interest as it suggested that the information in all 12 leads is needed to identify subtle patterns of ECG presentation across a larger population.

Given the size of the dataset (8,903 records) we opted to present a cross-validated result. Dataset 2 was randomly stratified into 10 folds (n=890 or n=891) with an even balance of sex within each age group over 10 year ranges. This gave 483 or 484 female records and 407 male records in each fold. We opted to retain all the data despite the small imbalance, although we did adapt the neural network second classifier to select only the same number of female records as male when training (selected initially at random and then retained for repeatability.)

To ensure independence of the test fold within the stacked model, each fold was treated as a full holdout set in turn. The remaining nine folds were used to generate a model and tune the parameters within a further nine-fold cross-validation, where seven folds were used for training, one for validation and one for testing in each cross-validated run. The holdout fold was then used to provide a classification accuracy for the model, and we report our final accuracy as the combined accuracies of each holdout fold (respecting the number of subjects in each fold). Supplement Figure 4 provides an overview of this process. Subject classification accuracy by holdout fold varied from 82.8% to 88.2%, despite the folds being balanced by sex and age. This indicates that presenting a cross-validated result provides a more comprehensive summary of the dataset, as one fold alone could give a skewed outcome.

We chose to present our classification results by accuracy and receiver operating characteristic (ROC) area under the curve (AUC) to allow comparison with other recent results in this space (1,12). Whilst simple accuracy can be a problematic metric when reporting many classification problems due to differing prevalence of the classes, it is an appropriate and easily understood metric in the case of a binary classification of sex.

Optimization of parameters and hyperparameters within the process was undertaken at various stages, using prior experience of our work in this space, grid searches and Bayesian optimization techniques. The final parameter details and the ranges considered are provided in Main Paper Figure 4. We made the decision to set parameters at a global level across the model (for example, just one k value for all k-NN models), rather than highly tuning each internal cross-validation loop, as we were aiming to create a more robust final model that could be more easily explained. The process of determining the global parameter choices was made by inspection of local optimization results. We appreciate that improvements could therefore be made to the model by considering different feature inputs and parameters by lead, and we look to undertake further work on this. Within the optimization process, we note that only using the original attractor method with $N = 3$ points on the signal achieves a classification accuracy of around 80%, which is the best result for a single choice of N.

We generated a final model using all the data in Dataset 2, and the parameters indicated by review of the optimization. All the Dataset 2 measures sets (r density, θ density, attractor outline r) were used to train the k-NN models. The posterior probability scores from the holdout folds of the cross-validated model were then applied to generate the lead and subject neural network models. Dataset 1 was then used as an unseen test set on this final model.

A frequent concern with machine learning models is the quality of the labelling of the data. In this case, we are confident of the accuracy of the female / male labels. In Dataset 2, the selection of normal records by taking those with the label 'Norm' could introduce mis-labelled recordings. We are confident that 7,442 of the 8,903 records are appropriately labelled as these have been validated by a human (8). There could be concern about the automated labelling of the remaining records, although we would expect that the identification of a normal state correctly is more likely than a disease state, so we accepted these records as provided.

SUPPLEMENT VIDEO CAPTIONS

Supplement Video I: Transforming an ECG signal into a 3-point attractor
The Symmetric Projection Attractor Reconstruction method transforms an entire digital signal into a corresponding two-dimensional image. In this animation, we show how we place three equally spaced points on the signal and then plot these values in three dimensions to create a different representation of the entire signal. By projecting this to a two dimensional image – our 'attractor' – we can visualize and quantify the morphology and variability of the underlying signal.

Supplement Video II: The three attractors associated with *N* = 7 points
In this example of the Symmetric Projection Attractor Reconstruction method, we embed the signal into seven dimensions. A seven-dimensional embedding cannot be visualized since we cannot plot in more than three dimensions. However, we can create three appropriate projected images ('attractors') that allow us to both visualize and quantify this higher dimensional object.

ADDITIONAL ACCOMPANYING FILES

The following files also accompany this Supplementary Material.

- **Graphical abstract**
  - *Graphical Abstract for Symmetric Projection Attractor Reconstruction Sex Differences in the ECG.jpeg*: A visual overview of this study.

- **Datasets**: Updated metadata .csv files for Dataset 1 and Dataset 2, ordered to match the subsequent analyses.
  - *Detecting Sex with SPAR_Dataset 1 Ordered.csv*: Records for Dataset 1 with accompanying metadata. All metadata is as provided with the original data, and none was added for this study.
  - *Detecting Sex with SPAR_Dataset 2 Ordered.csv*: Records for Dataset 2 with accompanying metadata. The columns added for this study (based on the original metadata) were TYPE, PERC, TYPEPERC, ORDER, NORMORDER, STRAT10NORM.
    The pertinent variables for this study are:
    - TYPE: Selected to be 'NORM'
    - NORMORDER: Selected to be 'FIRST'
    - STRAT10NORM: A random stratification (numbered 1 to 10) of the selected records to be balance by age and sex.

- **Average cycle length (ACL)**.
  - *Detecting Sex with SPAR_Dataset 2 ACL Update.csv*: Contains details of the 18 records of Dataset 2 that failed the ACL validation and required manual update.

The following files are also available from the authors.

- **Average cycle length (code)**: Matlab code to provide the ACL for a 12 lead ECG signal under the validation applied for this study. The function to determine the RR intervals is based on (10) and makes use of Matlab code detailed in (11).

- **Classification (code and results)**
  - *Classification code*: Matlab code used for the cross-validation model developed with Dataset 2.

- *Holdout_result.mat*: The classification scores of the cross-validation of Dataset 2. The results are stored by holdout fold and appear in the order of the records as given in DATASETS / *Dataset 2 Ordered.csv*.
- *Unseen_result.mat*: The classification scores of the cross-validation of Dataset 1. The results are stored by holdout fold and appear in the order of the records as given in DATASETS / *Dataset 1 Ordered.csv*.

Please note that Philip J. Aston and Manasi Nandi have a patent (WO2015121679A1 "Delay coordinate analysis of periodic data"), which covers the foundations of the SPAR method used in this paper and therefore any code content relating to this cannot be shared in a public domain. However, further files may be made available on request and we welcome questions and collaboration.

Corresponding author: Philip J. Aston, Department of Mathematics, University of Surrey, Guildford GU2 7XH, UK. Email: p.aston@surrey.ac.uk.

References for Supplementary Material (all references are in the main paper)

1. Attia ZI, Friedman PA, Noseworthy PA, Lopez-Jimenez F, Ladewig DJ, Satam G, et al. Age and sex estimation using artificial intelligence from standard 12-Lead ECGs. *Circ Arrhythmia Electrophysiol*. 2019;12(9):1–11.
2. Goldberger AL, Amaral LAN, Glass L, Hausdorff JM, Ivanov PC, Mark RG, et al. PhysioBank, PhysioToolkit, and PhysioNet components of a new research resource for complex physiologic signals. *Circulation*. 2000;101(23):e215–20.
3. Johannesen L, Vicente J, Mason JW, Sanabria C, Hong M, Guo P, et al. Differentiating drug-induced multichannel block on the electrocardiogram: Randomized study of dofetilide, quinidine, ranolazine, and verapamil. *Clin Pharmacol Ther*. 2014;96(5):549–58.
4. Vicente J, Johannesen L, Mason JW, Crumb WJ, Pueyo E, Stockbridge N, et al. Comprehensive T wave morphology assessment in a randomized clinical study of dofetilide, quinidine, ranolazine, and verapamil. *J Am Heart Assoc*. 2015;4:e001615.
5. Johannesen L, Vicente J, Mason J, Erato C, Sanabria C, Waite-Labott K, et al. Late sodium current block for drug-induced long QT syndrome: Results from a prospective clinical trial. *Clin Pharmacol Ther*. 2016;99(2):214–23.
6. Vicente J. Update on the ECG component of the CiPA initiative. *J Electrocardiol*. 2018;51(6):S98–102.
7. Moss AJ. Gender Differences in ECG Parameters and Their Clinical Implications. *Ann Noninvasive Electrocardiol*. 2010;15(1):1–2.
8. Wagner P, Strodthoff N, Bousseljot RD, Kreiseler D, Lunze FI, Samek W, et al. PTB-XL, a large publicly available electrocardiography dataset. *Sci Data*. 2020;7(1):1–15.
9. Aston PJ, Christie MI, Huang YH, Nandi M. Beyond HRV: Attractor reconstruction using the entire cardiovascular waveform data for novel feature extraction. *Physiol Meas*. 2018;39:024001.
10. Pan J, Tompkins WJ. A real-time QRS detection algorithm. *IEEE Trans Biomed Eng*. 1985;BME-32(3):230–6.
11. Sedghamiz H. Matlab implementation of Pan Tompkins ECG QRS detector [Internet]. MathWorks; 2014. Available from: https://fr.mathworks.com/matlabcentral/fileexchange/45840-complete-pan-tompkins-implementationecg-qrs-detector
12. Strodthoff N, Wagner P, Schaeffter T, Samek W. Deep learning for ECG analysis: Benchmarks and insights from PTB-XL. *IEEE J Biomed Heal Informatics*. 2021;25(5).
13. Stevens LM, Mortazavi BJ, Deo RC, Curtis L, Kao DP. Recommendations for reporting machine learning analyses in clinical research. *Circ Cardiovasc Qual Outcomes*. 2020;(October):782–93.
14. Lyle J V, Charlton PH, Bonet-Luz E, Chaffey G, Christie M, Nandi M, et al. Beyond HRV: Analysis of ECG signals using attractor reconstruction. In: Computing in Cardiology. 2017.
15. Aston PJ, Lyle J V., Bonet-Luz E, Huang CLH, Zhang Y, Jeevaratnam K, et al. Deep learning applied to attractor images derived from ECG signals for detection of genetic mutation. In: Computing in Cardiology. 2019.
16. Bonet-Luz E, Lyle J V., Huang CL-H, Zhang Y, Nandi M, Jeevaratnam K, et al. Symmetric Projection Attractor Reconstruction analysis of murine electrocardiograms: Retrospective prediction of Scn5a+/- genetic mutation attributable to Brugada syndrome. *Heart Rhythm O2*. 2020;1(5):368–75.
17. Lyle J, Aston P, Nandi M. Investigating the response to dofetilide with Symmetric Projection Attractor Reconstruction of the electrocardiogram. In: Computing in Cardiology. 2019.
18. Wolpert DH. Stacked generalization. *Neural Networks*. 1992;5(2):241–59.

Table 1: Characteristics of the data in Datasets 1 and 2. The median and inter-quartile range (IQR) are provided as the data would be expected to be non-gaussian. We follow the justification of Vicente et al (6) in correcting the QT and JTpeak intervals for heart rate, taking Friderica's correction for QT ($QTc = QT/RR^{1/3}$ with RR in seconds) and a correction to JT_{peak}, ($JT_{peak}c = JT_{peak}/RR^{0.58}$ with RR in seconds), whilst making no correction in the $T_{peak}T_{end}$ interval, as prior studies suggest rate independence at resting heart rates.

	**Female**	**Male**
**Dataset 1**		
No. of subjects	42	62
Age (years)	27 (23, 31)	29 (23, 35)
Height (cm)	163 (161, 167)	176 (174, 181)
Weight (kg)	68 (63, 72)	76 (69, 84)
**Intervals** (ms)		
RR	921 (862, 1006)	1093 (995, 1197)
PR	164 (150, 178)	166 (155, 180)
QRS	87 (81, 90)	90 (83, 100)
QTc	402 (389, 413)	385 (376, 395)
$JT_{peak}c$	248 (228, 257)	222 (206, 230)
$T_{peak}T_{end}$	70 (67, 75)	73 (65, 82)
**Dataset 2**		
No. of subjects	4,833	4,070
Age (years)	53 (40, 66)	54 (41, 64)

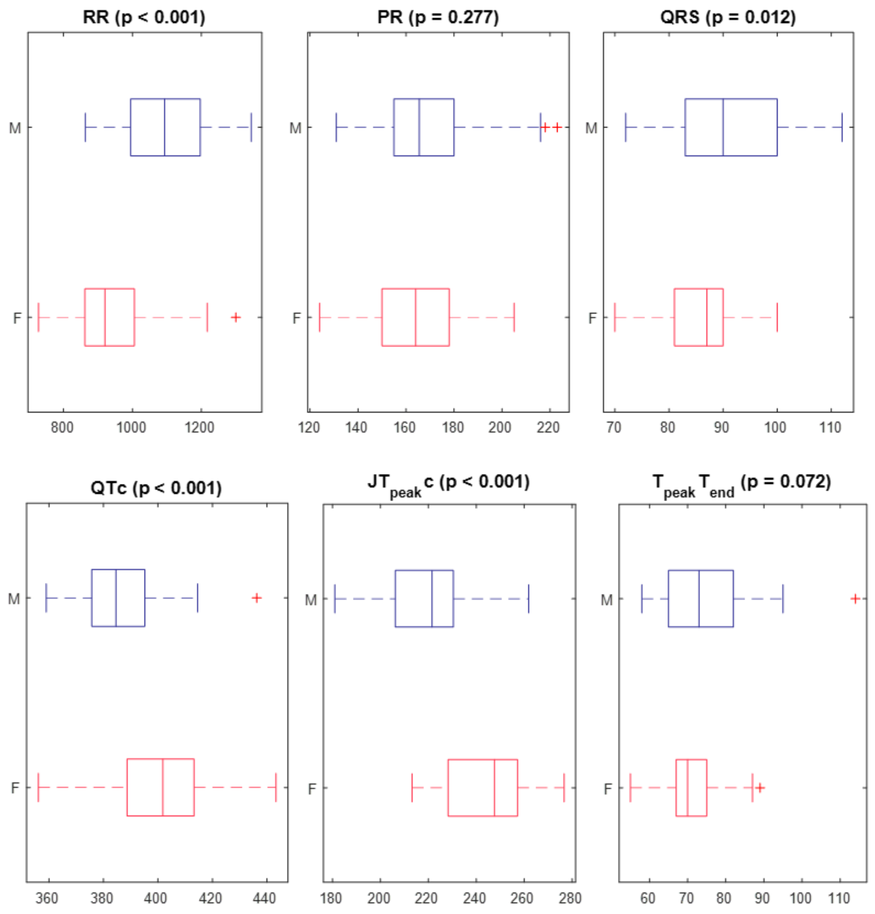

Figure 1: Boxplots for interval measures of Dataset 1. Plots are shown for male (blue) and female (red.) A significance test for the difference in median values was determined by the Mann-Whitney U test, with p-values as shown, indicating a significant difference in RR, QTc and JTpeakc at the 1% level between males and females.

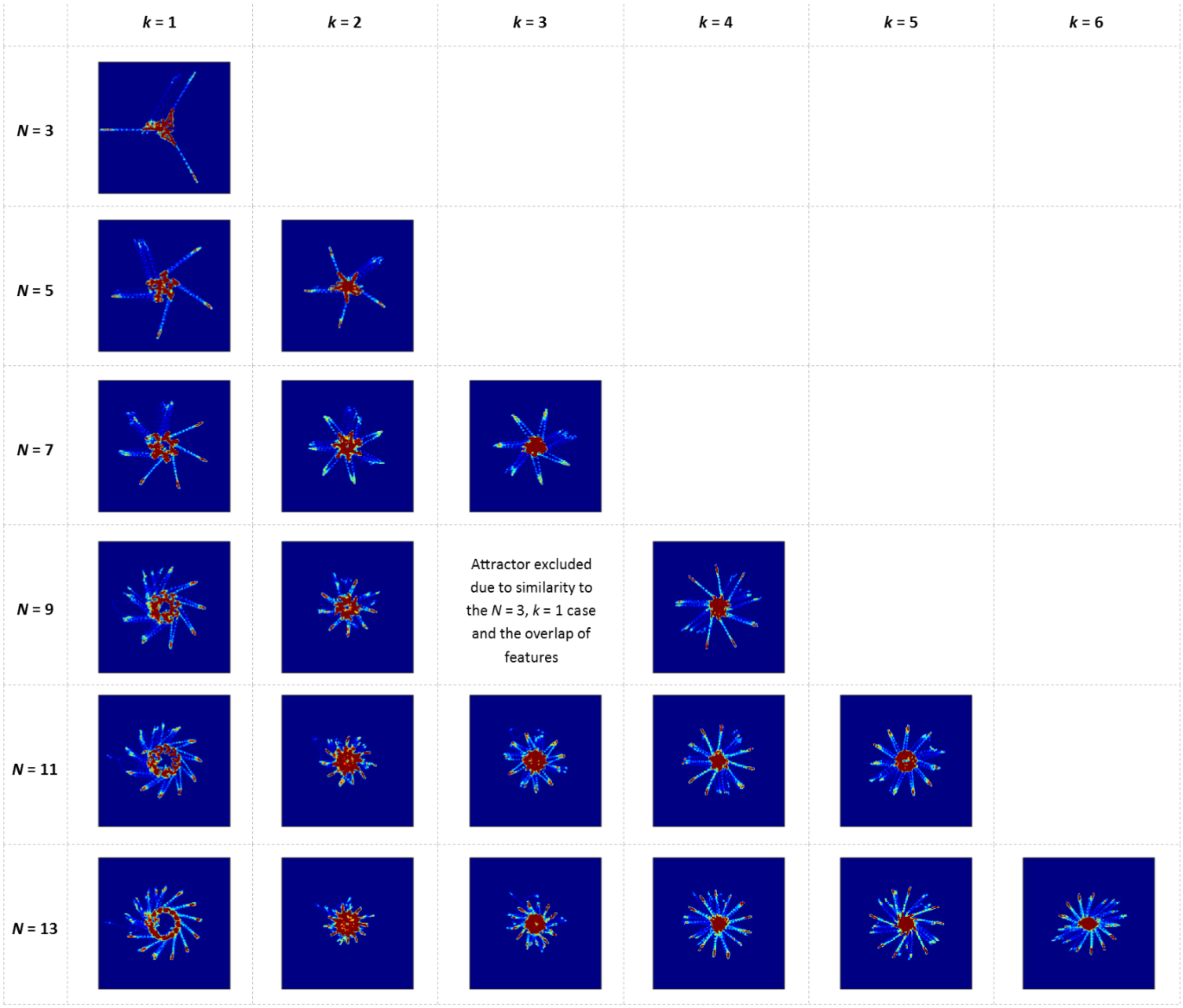

Figure 2: Attractors generated from a lead II signal for this study. Attractors are generated from the male lead II signal shown in Main Paper Figure 2 using all odd numbers of points N = 3, 5, ..., 13. As N increases, the number of possible projections k to a two-dimensional image also increases. We note that for the specific case N = 9, k = 3, we do not generate an attractor due to its similarity to the appearance of N = 3, k = 1.

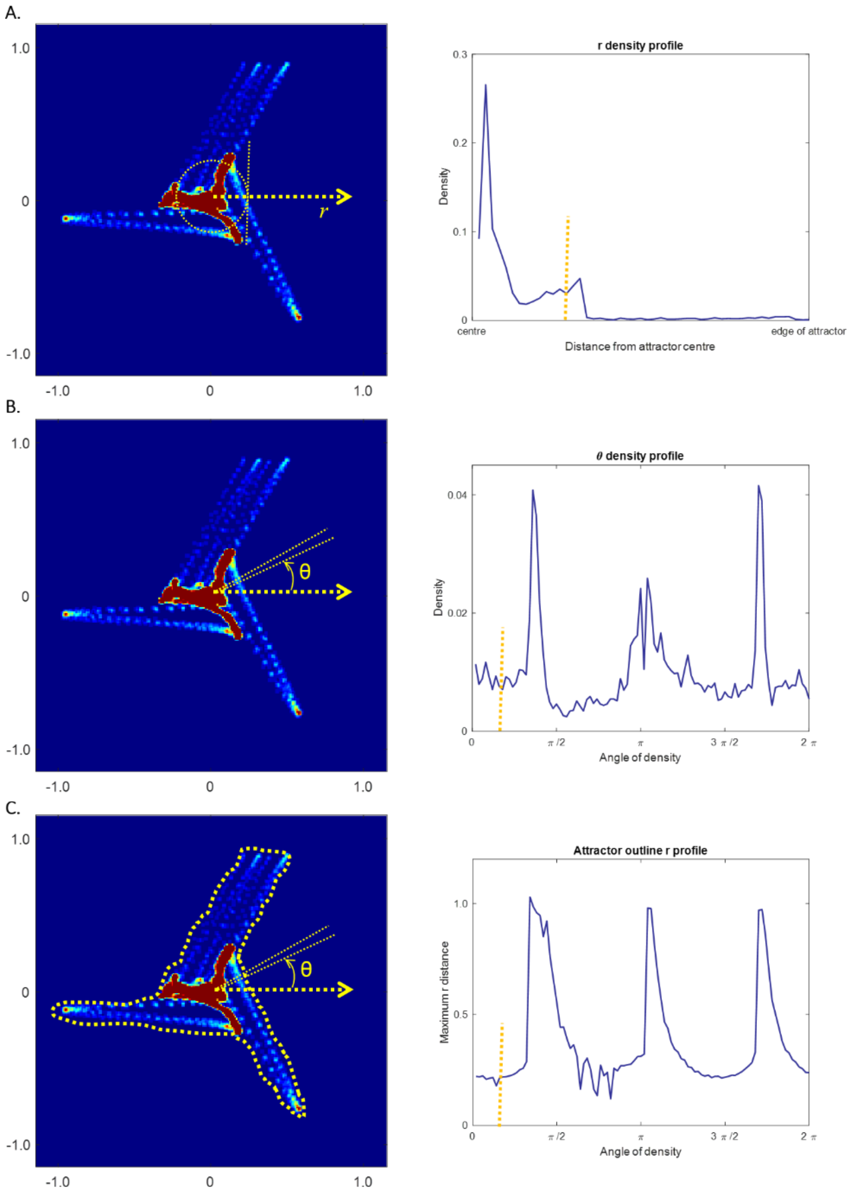

Figure 3: How the three measure sets are determined for an attractor. The attractor image is a 3-point attractor from the female lead II signal shown in Main Paper Figure 1. (**A**) The radial density distribution (r density) based on the distance from the centre to the outer edge of the attractor. We divide the distance from the centre to the outer edge of each attractor into 50 bins and simply sum up the number of points falling into each bin to give one distribution of the density of the attractor. (**B**) The θ density distribution (θ density.) We apply a similar approach in the angular direction by sweeping around the attractor in a circular direction and dividing the 360 degrees traversed into 100 bins and again summing up the number of points in each bin to give an alternative distribution of the density of the attractor. (**C**) The outline shape of the image as maximum r in the θ direction (attractor outline r.) We combine the radial and angular measures by taking the maximum distance of the attractor from the centre for each of 100 angular bins.

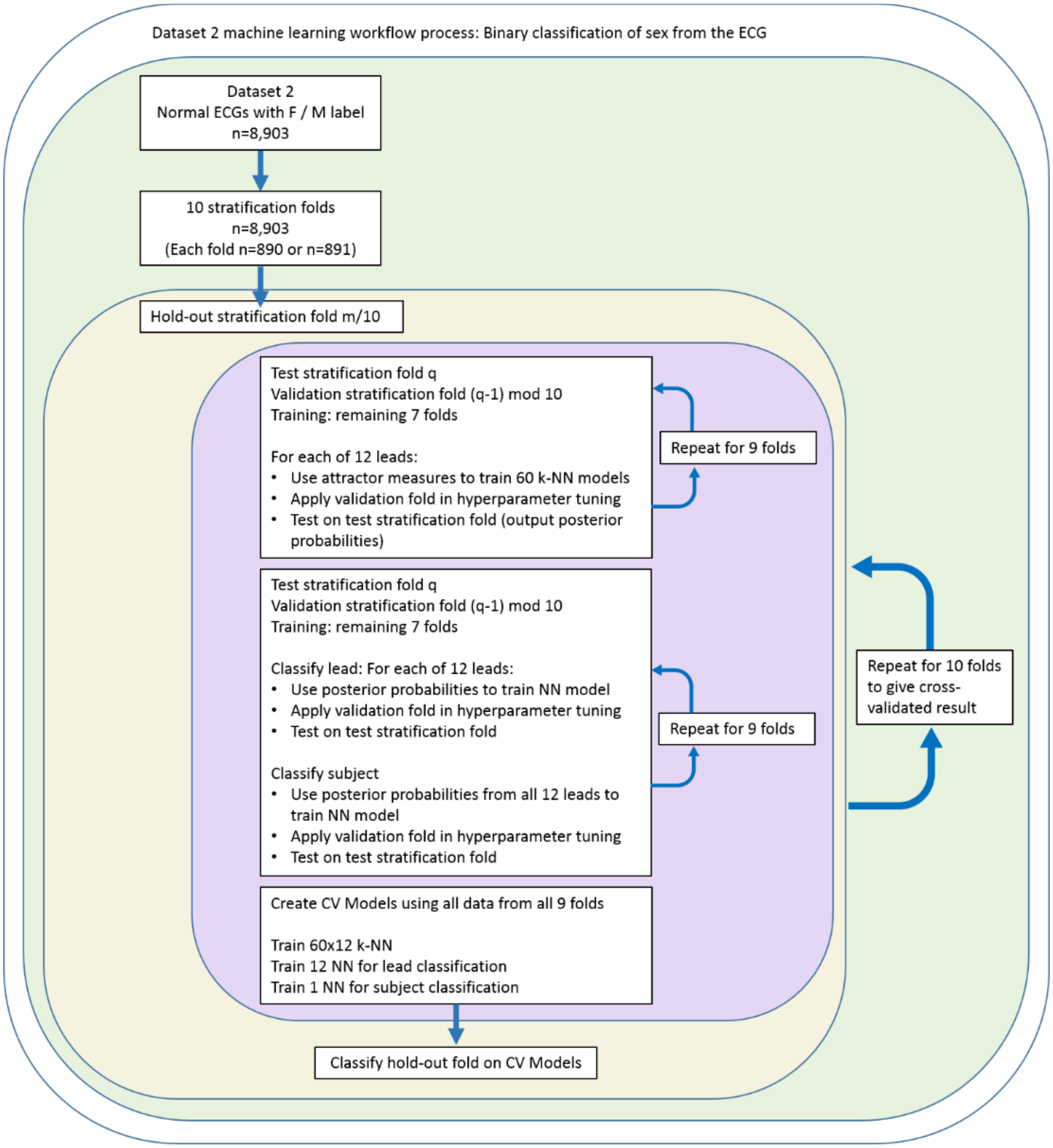

Figure 4: Workflow process for the cross-validated model created with Dataset 2.
