# Supplementary figures and images for: Symmetric Projection Attractor Reconstruction: Sex Differences in the ECG
Source: Front Cardiovasc Med. 2021 Sep 23;8:709457. doi: 10.3389/fcvm.2021.709457 (PMC8495026; doi:10.3389/fcvm.2021.709457)

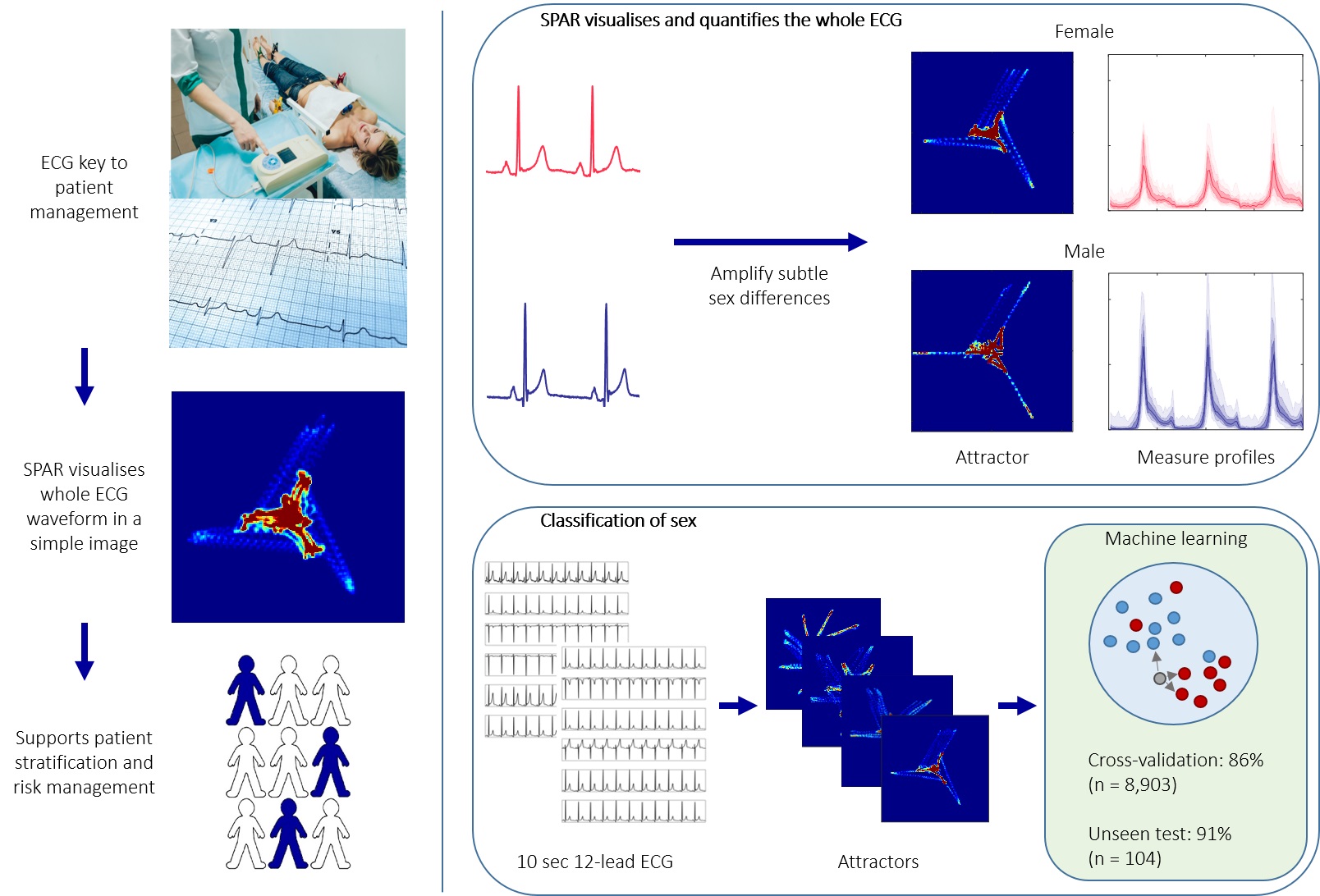

Supplement: Supplementary file 1 [file Data_Sheet_1.zip › Graphical Abstract for Symmetric Projection Attractor Reconstruction Sex Differences in the ECG.jpg]
